# Whole genome de novo sequencing and comparative genomic analyses suggests that *Chlamydia psittaci* strain 84/2334 should be reclassified as *Chlamydia abortus* species

**DOI:** 10.1186/s12864-021-07477-6

**Published:** 2021-03-06

**Authors:** David Longbottom, Morag Livingstone, Paolo Ribeca, Delphine Sylvie Anne Beeckman, Arie van der Ende, Yvonne Pannekoek, Daisy Vanrompay

**Affiliations:** 1 Moredun Research Institute, Pentlands Science Park, Bush Loan, Edinburgh, Midlothian, EH26 0PZ UK; 2 Biomathematics and Statistics Scotland, Peter Guthrie Tait Road, Edinburgh, EH9 3FD UK; 3 Department of Molecular Biotechnology, Faculty of Bioscience Engineering, University of Ghent, Ghent, Belgium; 4 Current address: BASF Belgium Coordination Center CommV – Innovation Center Gent, Ghent, Belgium; 5 Department of Medical Microbiology, Amsterdam UMC, University of Amsterdam, Amsterdam, The Netherlands; 6 Department of Animal Science and Aquatic Ecology, Faculty of Bioscience Engineering, University of Ghent, Ghent, Belgium

**Keywords:** *Chlamydia abortus*, *Chlamydia psittaci*, Genome sequence, Phylogenomics, Comparative genomic analysis, Polymorphic membrane proteins, Plasticity zone, MLST

## Abstract

**Background:**

*Chlamydia abortus* and *Chlamydia psittaci* are important pathogens of livestock and avian species, respectively. While *C. abortus* is recognized as descended from *C. psittaci* species, there is emerging evidence of strains that are intermediary between the two species, suggesting they are recent evolutionary ancestors of *C. abortus*. Such strains include *C. psittaci* strain 84/2334 that was isolated from a parrot. Our aim was to classify this strain by sequencing its genome and explore its evolutionary relationship to both *C. abortus* and *C. psittaci*.

**Results:**

In this study, methods based on multi-locus sequence typing (MLST) of seven housekeeping genes and on typing of five species discriminant proteins showed that strain 84/2334 clustered with *C. abortus* species. Furthermore, whole genome de novo sequencing of the strain revealed greater similarity to *C. abortus* in terms of GC content, while 16S rRNA and whole genome phylogenetic analysis, as well as network and recombination analysis showed that the strain clusters more closely with *C. abortus* strains. The analysis also suggested a closer evolutionary relationship between this strain and the major *C. abortus* clade, than to two other intermediary avian *C. abortus* strains or *C. psittaci* strains. Molecular analyses of genes (polymorphic membrane protein and transmembrane head protein genes) and loci (plasticity zone), found in key virulence-associated regions that exhibit greatest diversity within and between chlamydial species, reveal greater diversity than present in sequenced *C. abortus* genomes as well as similar features to both *C. abortus* and *C. psittaci* species. The strain also possesses an extrachromosomal plasmid, as found in most *C. psittaci* species but absent from all sequenced classical *C. abortus* strains.

**Conclusion:**

Overall, the results show that *C. psittaci* strain 84/2334 clusters very closely with *C. abortus* strains, and are consistent with the strain being a recent *C. abortus* ancestral species. This suggests that the strain should be reclassified as *C. abortus*. Furthermore, the identification of a *C. abortus* strain bearing an extra-chromosomal plasmid has implications for plasmid-based transformation studies to investigate gene function as well as providing a potential route for the development of a next generation vaccine to protect livestock from *C. abortus* infection.

**Supplementary Information:**

The online version contains supplementary material available at 10.1186/s12864-021-07477-6.

## Introduction

The family *Chlamydiaceae* comprises a group of obligate intracellular Gram-negative bacteria that are responsible for a broad range of infections in non-human mammals, birds and humans [1]. Since 1999 the family has undergone a number of taxonomic reclassifications at the genus and species levels based on sequence analysis of the 16S and 23S rRNA genes, as well as taking into account biological differences [2–5]. Current classification within this family recognises a single genus, *Chlamydia*, and 14 species (*Chlamydia abortus, Chlamydia avium, Chlamydia buteonis, Chlamydia caviae, Chlamydia felis, Chlamydia gallinacea, Chlamydia muridarum, Chlamydia pecorum, Chlamydia pneumoniae, Chlamydia poikilothermis, Chlamydia psittaci, Chlamydia serpentis, Chlamydia suis* and *Chlamydia trachomatis*) [6–8], plus a further four *Candidatus* species (Ca. Chlamydia ibidis, Ca. Chlamydia corallus*,* Ca. Chlamydia sanzinia and Ca. Chlamydia testudinis) [9–12].

One of the oldest recognised chlamydial species is *C. psittaci* that is associated with respiratory, ocular and enteric infections in psittacine birds and poultry [1, 13]. Shedding of the pathogen in faecal, respiratory and ocular secretions can occur and result in zoonotic infections in humans, leading to pneumonia (psittacosis) that can be fatal [13, 14]. Such infections have been frequently reported in veterinarians, breeders, pet shop and poultry workers [1, 14]. *C. psittaci* has been classified into nine genotypes based on *ompA* (major outer membrane protein gene) sequencing [15], of which seven (A-F, E/B) are each generally associated with specific species of birds, while a further two were isolated from mammalian species, specifically cattle (WC) and muskrat (M56) [16]. In addition, a further ten provisional genotypes (1V, Mat116, YP84, R54, 6N, CPX0308, I, J, G, G1 and G2) have been described [17–19]. More recently, three newly described species, *C. gallinacea*, *C. avium* and *C. buteonis*, have also been found in birds [11, 20], with *C. buteonis* suggested to have a phylogenetic intermediate position between *C. psittaci* and *C. abortus* species [7].

Chlamydial infections in non-avian livestock species are principally caused by three species, *C. abortus*, *C. pecorum* and *C. suis* [1], of which *C. abortus* is considered the most closely related to and suggested to be evolved from *C. psittaci* species [21]. *C. abortus* is principally responsible for causing enzootic abortion (syn. ovine enzootic abortion, OEA; enzootic abortion of ewes, EAE; ovine chlamydiosis) in sheep and goats [1, 22]. This organism, which was first described in 1950 [23], generally causes abortion in the last 2 to 3 weeks of pregnancy or the birth of weak or stillborn lambs or kids. *C. abortus* also causes sporadic reproductive failure in cattle, horses and pigs and the bacterium presents a dangerous zoonotic risk to pregnant women, in whom it can cause spontaneous abortion, as well as being potentially fatal for the woman [24–26]. In contrast to *C. psittaci*, the genome of *C. abortus* is considered to be relatively stable with very little diversity and undergoing little or no recombination [27].

It has long been recognised that there are a number of *C. psittaci* strains that differ from the classical avian *C. psittaci* strains, with comparative sequence and MLST analysis suggesting that they are more closely related to *C. abortus* species [21, 28]. Such strains have been classified as belonging to genotype F by *ompA Alu*I typing, and include isolates Prk/Daruma (isolated from parakeets) [29], 10,433-MA (isolated from a parrot) [30], VS225 (isolated from a parakeet) [2], and 84/2334 (isolated from a yellow-crowned Amazon parrot) [31]. These isolates, as well as others recently described with provisional genotypes 1V, G1 and G2 [18], have been variously referred to as atypical *C. psittaci/C. abortus*, *C. psittaci*/*C.abortus* intermediate and avian *C. abortus* strains [21]. The purpose of this study was to characterise and sequence the genome of one of these atypical *C. psittaci* strains, specifically strain 84/2334, and determine its similarity and genetic relatedness to both *C. psittaci* and *C. abortus* species through comparative sequence analysis.

## Results

### Molecular typing of *Chlamydia psittaci* strain 84/2334

**Fig. 1:**
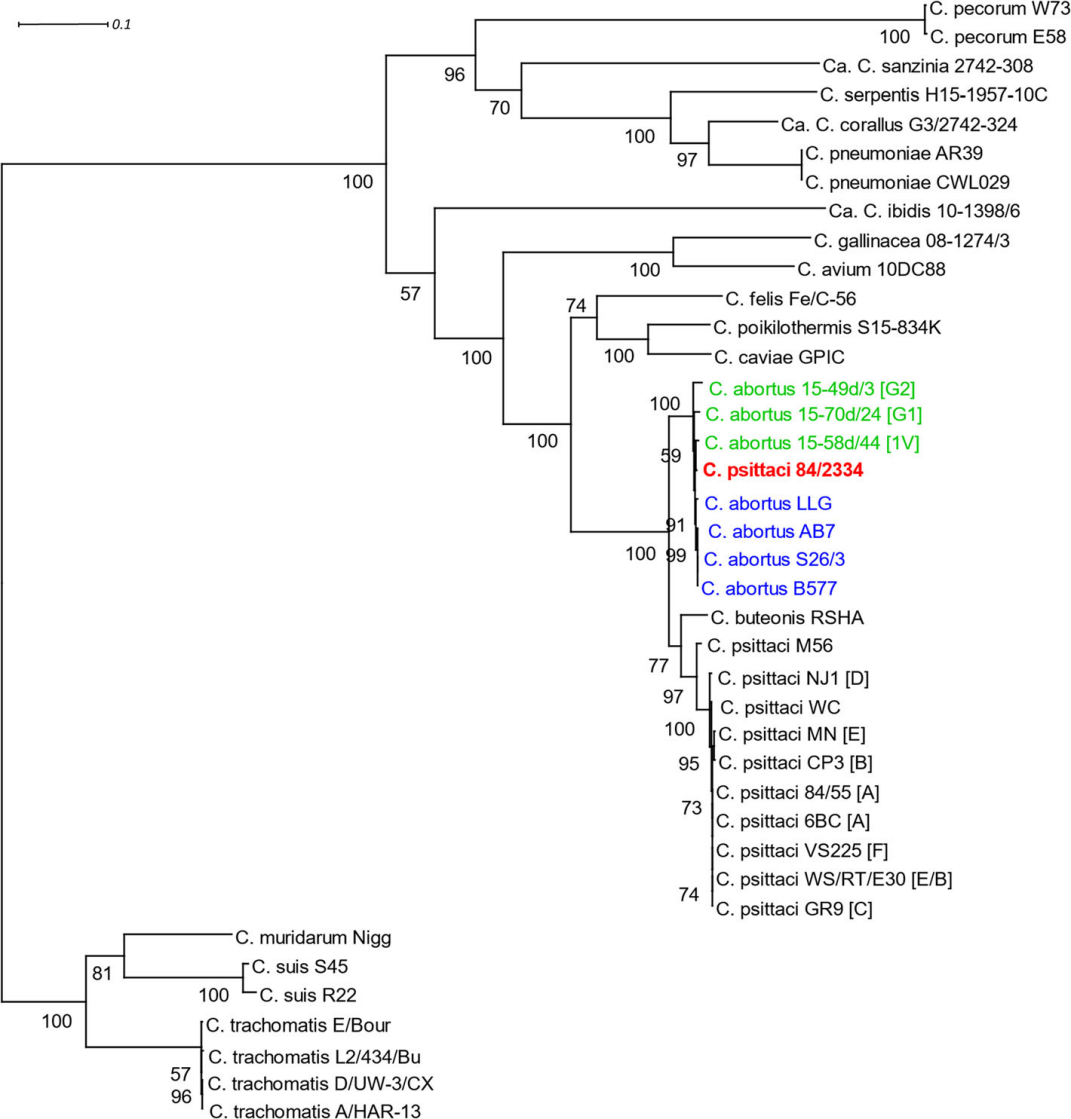
MLST phylogenetic tree for *C. psittaci* strain 84/2334 and representative strains of *Chlamydiaceae* species. The consensus PhyML phylogenetic tree of a 3147 bp alignment of concatenated sequences of seven MLST housekeeping gene fragments *(enoA*, *fumC*, *gatA*, *gidA*, *hemN*, *hlfX* and *oppA*) was estimated in TOPALi by Maximum Likelihood using a GTR + G + I substitution and rate heterogeneity model, according to BIC model selection, and 100 non-parametric bootstrap replicates. The tree is rooted on the *C. trachomatis*/*muridarum*/*suis* branch and bootstrap support is indicated by the number at the node. The scale bar indicates the expected substitutions per site. Genotypes are given in square brackets. The tree was prepared for publication in Dendroscope. Strain 84/2334 is in bold and red font. Classical and avian *C. abortus* strains are in blue and green fonts, respectively

PhyML phylogenetic analysis of a 3147 bp alignment of concatenated fragments of seven multi-locus sequence typing (MLST) housekeeping genes (*enoA*, *fumC*, *gatA*, *gidA*, *hemN*, *hlfX* and *oppA*) [28, 32] for *C. psittaci* strain 84/2334 as well as other representative strains from each of the currently recognised *Chlamydiaceae* species are shown in Fig. 1. Strain 84/2334 was determined to cluster closely with the classical *C. abortus* strains, as previously observed using a less robust Neighbour-Joining method [28], as well as the three atypical avian *C. abortus* strains (15-70d/24, 15-49d/3 and 15-58d/44). Indeed, the strain appears more closely related to the classical *C. abortus* strains than to the avian *C. abortus* or *C. psittaci* strains, which is further supported by in silico genome-to-genome distance comparison [33] with published reference *C. abortus* and *C. psittaci* genomes (Additional file 1). In addition, it was noted that *C. buteonis* strain RSHA appears to cluster with *C. psittaci* species, but having a phylogenetic position somewhere between *C. psittaci* and *C. abortus*, as previously reported [7]. Cluster analysis of the allelic profiles or sequence type (ST) of each strain in minimum spanning trees produced a similar result (Additional file 2), with strain 84/2334 clustering with the *C. abortus* strains. Phylogenetic analysis of the 16S rRNA sequences also produced a similar tree structure to that obtained by MLST analysis (Additional file 3), the only exception being *C. buteonis* strain RSHA which clusters with *C. abortus* species, while ribosomal MLST (rMLST) based on 53 genes encoding the bacterial ribosome protein subunits (*rps* genes) [34] predicted the species taxon of strain 84/2334 as *C. abortus* with 100% support (Additional file 4).

Utilising another typing system developed by Pillonel et al. [35] the protein sequences of five highly divergent proteins (Adk, FtsK, HemL, PepF and RpoN) used for distinguishing species were extracted for strain 84/2334 from the MLST database [36]. These sequences were compared to those of representative *C. abortus* (S26/3, AB7, LLG, 15-70d/24, 15-49d/3 and 15-58d/44) and *C. psittaci* (6BC, CP3, GR9, NJ1, MN, VS225, WS/RT/E30, M56 and WC) species and genotypes to determine the percentage sequence identity. Comparison of these sequence similarities for all five discriminant proteins with the threshold scores for distinguishing strains at the species level suggest that strain 84/2334 should be classified as *C. abortus* (Table 1).

**Table 1:** Species classification of *C. psittaci* strain 84/2334 based on similarity of five discriminant protein sequences

	Percentage sequence similarity to equivalent protein in ***C. psittaci*** strain 84/2334														
	*C. abortus* strains			Avian *C. abortus* strains			*C. psittaci* strains								
	S26/3	AB7	LLG	15-70d/24 [G1] ^1^	15-49d/3 [G2] ^1^	15-58d/44 [1V] ^1^	6BC [A] ^1^	CP3 [B] ^1^	GR9 [C] ^1^	NJ1 [D] ^1^	MN [E] ^1^	VS225 [F] ^1^	WS/RT/E30 [E/B] ^1^	M56	WC
Adk	**99.1**	**99.1**	**99.5**	**98.6**	**95.8**	NA	92.5	92.5	92.5	92.5	92.5	92.5	92.5	92.0	92.5
FtsK	**99.4**	**99.4**	**99.4**	**99.5**	**98.9**	**99.3**	97.0	96.9	97.0	96.8	96.8	96.9	97.0	96.8	97.0
HemL	**99.3**	**99.3**	**98.9**	**99.3**	**99.1**	**99.1**	92.2	92.2	92.4	92.0	92.4	92.4	92.4	92.2	92.4
PepF	**99.7**	**99.7**	**99.5**	**98.8**	**98.4**	**99.3**	94.1	93.9	94.1	94.2	94.1	94.2	94.1	94.1	94.4
RpoN	**99.8**	**98.8**	**99.1**	**98.1**	**98.1**	**98.8**	94.3	94.3	94.1	94.3	94.3	94.1	93.9	94.3	94.1

Species classification based on typing scheme of Pillonel et al. [35]. Numbers in bold indicate those that meet threshold criteria (Adk ≥ 95%, FtsK ≥98%, HemL ≥95%, PepF ≥96%, RpoN ≥96%) for speciation of strain 84/2334 as *C. abortus*. ^1^ Letters/numbers in square brackets indicate designated genotypes. NA, indicates not available in Genbank database

### Genomic sequencing of strain 84/2334

**Fig. 2:**
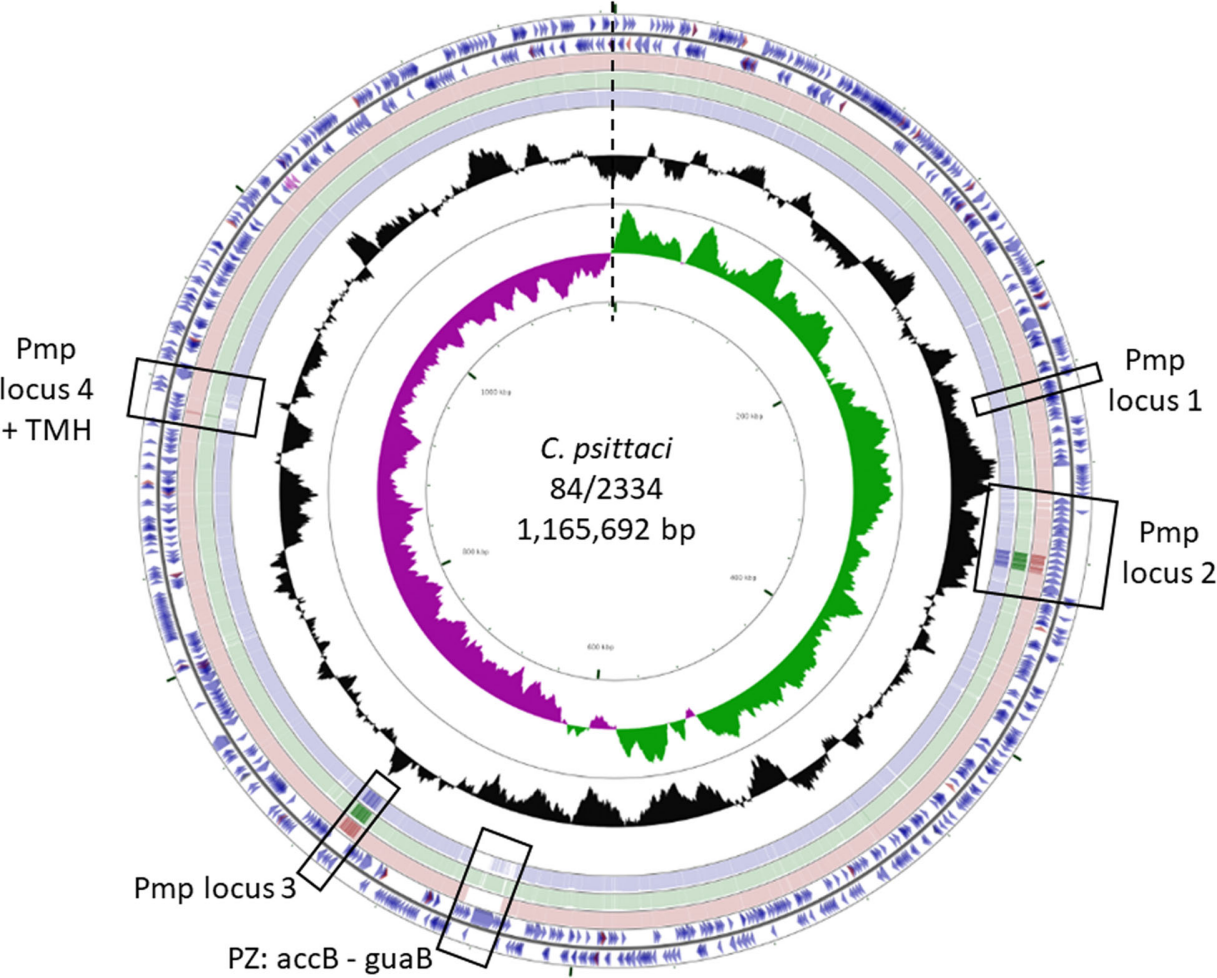
Circular representation of the genome of *C. psittaci* strain 84/2334. Circles from the outside in show: the positions of protein-coding genes (blue), tRNA genes (orange) and rRNA genes (Pink) on the positive (circle 1) and negative (circle 2) strands, respectively. Circles 3–5 shows positions of BLAST hits determined through blastn comparisons of *C. abortus* S26/3 (circle 3), *C. psittaci* 6BC (circle 4) and *C. psittaci* GR9 (circle 5) with the following settings: query split size = 50,000 bp, query split overlap size = 0, expect value cut off = 0.00001. Low complexity sequences were eliminated from the analysis. The height of the shading in the BLAST results rings is proportional to the percent identity of the hit. Overlapping hits appear as darker shading. Circles 6 and 7 show plots of the GC content and GC skew plotted as the deviation from the average for the entire sequence. The origin of replication is indicated by the vertical dashed line. The four Pmp loci, PZ region and TMH loci are highlighted using rectangular boxes. The figure was generated using the program CGView

 Genome sequencing and de novo assembly of *C. psittaci* strain 84/2334 successfully resulted in a single chromosomal contig with a 273x sequence coverage. The general features of the assembled genome in comparison to reference strains from *C. abortus* and *C. psittaci* species are shown in Table 2. The genome of *C. psittaci* strain 84/2334 comprises a single circular chromosome of 1,165,692 bp (Fig. 2) and in common with all other sequenced *C. psittaci* and *C. abortus* strains, 84/2334 has a single rRNA operon, 38 tRNA genes corresponding to all the amino acids except selenocysteine and pyrrolysine and a similar number of predicted coding sequences. Of note, the GC content of strain 84/2334 is 39.9%, which is similar to that of the *C. abortus* strains (Table 2).

**Table 2:** General genome features of strain 84/2334, compared with representative *C. psittaci* and *C. abortus* strains

Species	Strain	Genotype	Host	Chromosome Length (bp)	CDS ^**a**^	%GC ^**b**^	rRNA ^**c**^	tRNA ^**d**^	Plasmid ^**e**^ (length in bp)	Genbank Accession N^**o**^	
										Chromosome	Plasmid
*C. psittaci*	6BC	A	Parrot	1,171,667	987	39.1	1	38	Y (7,553)	CP002586.1	CP002587.1
*C. psittaci*	84/55	A	Amazon Parrot	1,172,064	1004	39.1	1	38	Y (7,487)	CP003790.1	CP003812.1
*C. psittaci*	CP3	B	Urban Pigeon	1,168,150	1002	39.1	1	38	Y (7,552)	CP003797.1	CP003813.1
*C. psittaci*	GR9	C	Mallard	1,147,152	1022	39.1	1	38	N	CP003791.1	None
*C. psittaci*	NJ1	D	Turkey	1,161,434	994	39.0	1	38	Y (7,552)	CP003798.1	CP003816.1
*C. psittaci*	MN	E	Human	1,168,490	993	39.1	1	38	Y (7,491)	CP003792.1	CP003815.1
*C. psittaci*	VS225	F	Parakeet	1,157,385	990	39.0	1	38	Y (7,553)	CP003793.1	CP003817.1
*C. psittaci*	WS/RT/E30	E/B	Mallard	1,140,789	980	39.0	1	38	Y (7,553)	CP003794.1	CP003819.1
*C. psittaci*	M56	M56	Muskrat	1,161,385	992	38.8	1	38	Y (7,553)	CP003795.1	CP003814.1
*C. psittaci*	WC	WC	Cattle	1,172,265	998	39.1	1	38	Y (7,553)	CP003796.1	CP003818.1
*C. abortus*	15-70d/24	G1	Eurasian Teal	1,141,702	993	39.8	1	38	Y (7,680)	LS450958.1	LS450959.1
*C. abortus*	15-49d/3	G2	Mallard	1,132,456	977	39.6	1	38	Y (7,683)	LS450956.1	LS450957.1
***C. psittaci***	**84/2334**	**F**	**Amazon Parrot**	**1,165,692**	**994**	**39.9**	**1**	**38**	**Y (7,553)**	**CP031646.1**	**CP031647.1**
*C. abortus*	S26/3 ^f^	wt	Sheep	1,144,377	970	39.9	1	38	N	CR848038.1	None
*C. abortus*	AB7 ^g^	wt	Sheep	1,144,467	971	39.9	1	38	N	LN554882.1	None
*C. abortus*	LLG ^h^	variant	Goat	1,143,694 ^i^	967	39.9	1	38	N	CP018296.1	None

^a^total number of predicted coding sequences; ^b^ percentage GC content of genomic sequence; ^c^ number of rRNA operons; ^d^ number of tRNA genes; ^e^ presence of extrachromosomal plasmid is indicted by Y (yes) or N (no) and if present length is given in brackets; ^f^ representative wild-type (wt) strain of UK origin [27]; ^g^ representative wt strain of French origin [37]; ^h^ one of two (identical) known variant strains of *C. abortus* [27, 38]; ^i^ genome incomplete (approx.. 4721 bp + 3457 bp missing in Pmp loci 2 and 3 (See Fig. 2), respectively). Sequence information for *C. psittaci* strain 84/2334 is highlighted in bold

**Fig. 3:**
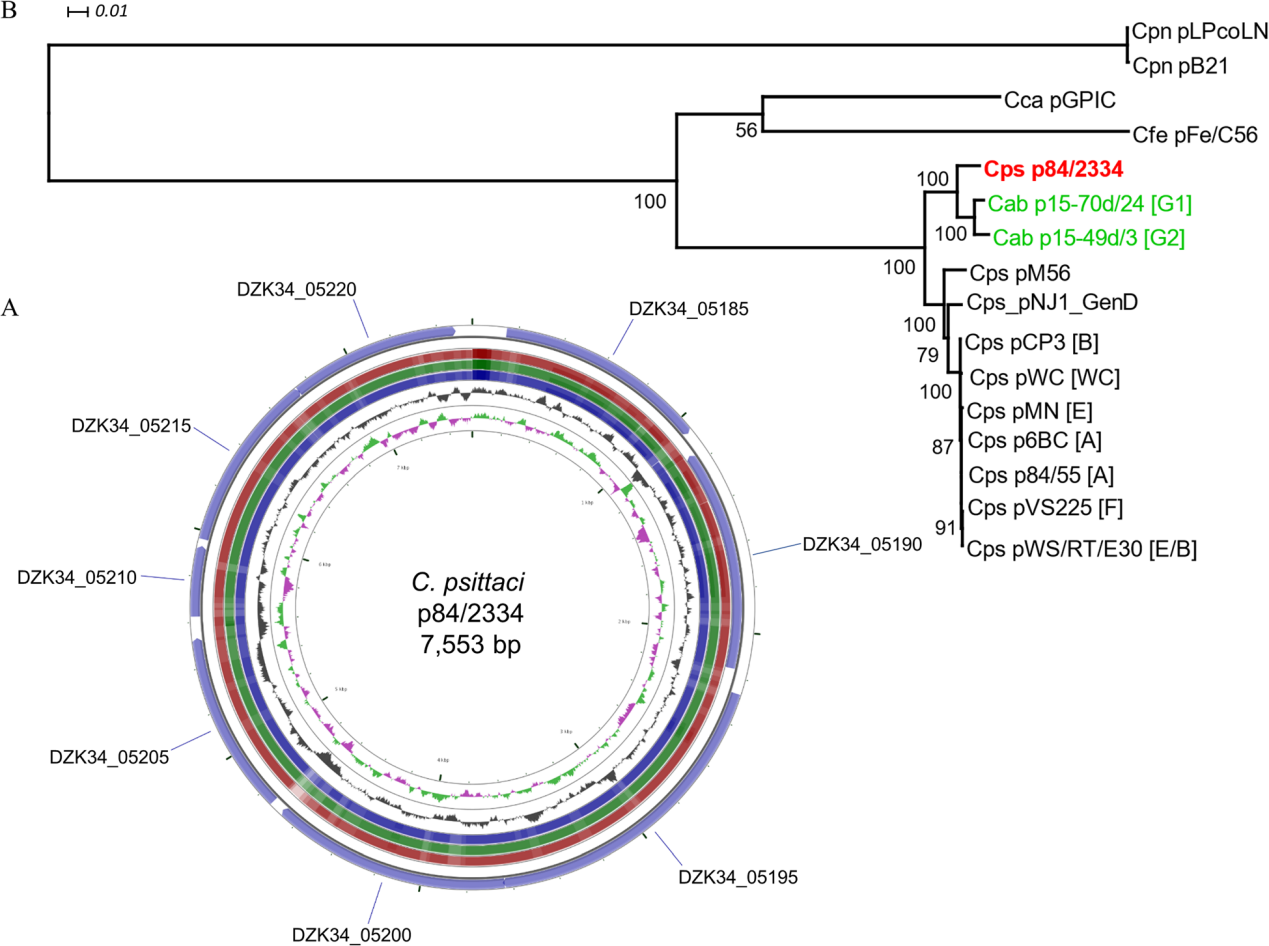
Circular representation of the *C. psittaci* 84/2334 plasmid sequence and comparative phylogenetic analysis. **a** Circular representation of the plasmid sequence of strain 84/2334; Circles from the outside in show: the positions of the eight coding sequences on the forward (circle 1) and reverse (circle 2) strands of p84/2334. Circles 3–5 show positions of BLAST hits determined through blastn comparisons of *C. psittaci* 6BC (circle 3), VS225 (circle 4) and M56 (circle 5) with the following settings: query split size = 50,000 bp, query split overlap size = 0, expect value cut off = 0.0001. The height of the shading in the BLAST results rings is proportional to the percent identity of the hit. Overlapping hits appear as darker shading. Circles 6 and 7 show plots of the GC content and GC skew plotted as the deviation from the average for the entire sequence. The figure was generated using the program CGView. **b** Phylogenetic tree of an alignment of the plasmid sequences of *C. psittaci* (Cps), avian *C. abortus* (Cab), *C. caviae* (Cca), *C. felis* (Cfe) and *C. pneumoniae* (Cpn) strains. The consensus tree was estimated in TOPALi by Maximum Likelihood (PhyML) using a TIM + G substitution and rate heterogeneity model, according to BIC model selection, and 100 non-parametric bootstrap replicates. The tree is midpoint rooted and bootstrap support is indicated by the number at the node. The scale bar indicates the expected substitutions per site. Genotypes are given in square brackets. The tree was prepared in Dendroscope. Strain 84/2334 plasmid is in bold and red font. Avian *C. abortus* strain plasmids are in green font

The sequence of the *C. psittaci* strain 84/2334 plasmid was extracted as a single contig from the total whole genome raw sequence reads by comparison with other *C. psittaci* plasmid sequences (Table 2). The 84/2334 plasmid comprises a single circular sequence of 7553 bp (Fig. 3a) with eight coding sequences, similar to those found in other sequenced plasmid-bearing chlamydial species [39, 40]. Phylogenetic analysis of the 84/2334 plasmid with other sequenced chlamydial plasmids (including *C. psittaci*, *C. penumoniae*, *C. felis* and *C. caviae*) shows that it clusters with those of the two avian *C. abortus* strains (Fig. 3b).

### Whole genome recombination and phylogenetic analysis

**Fig. 4:**
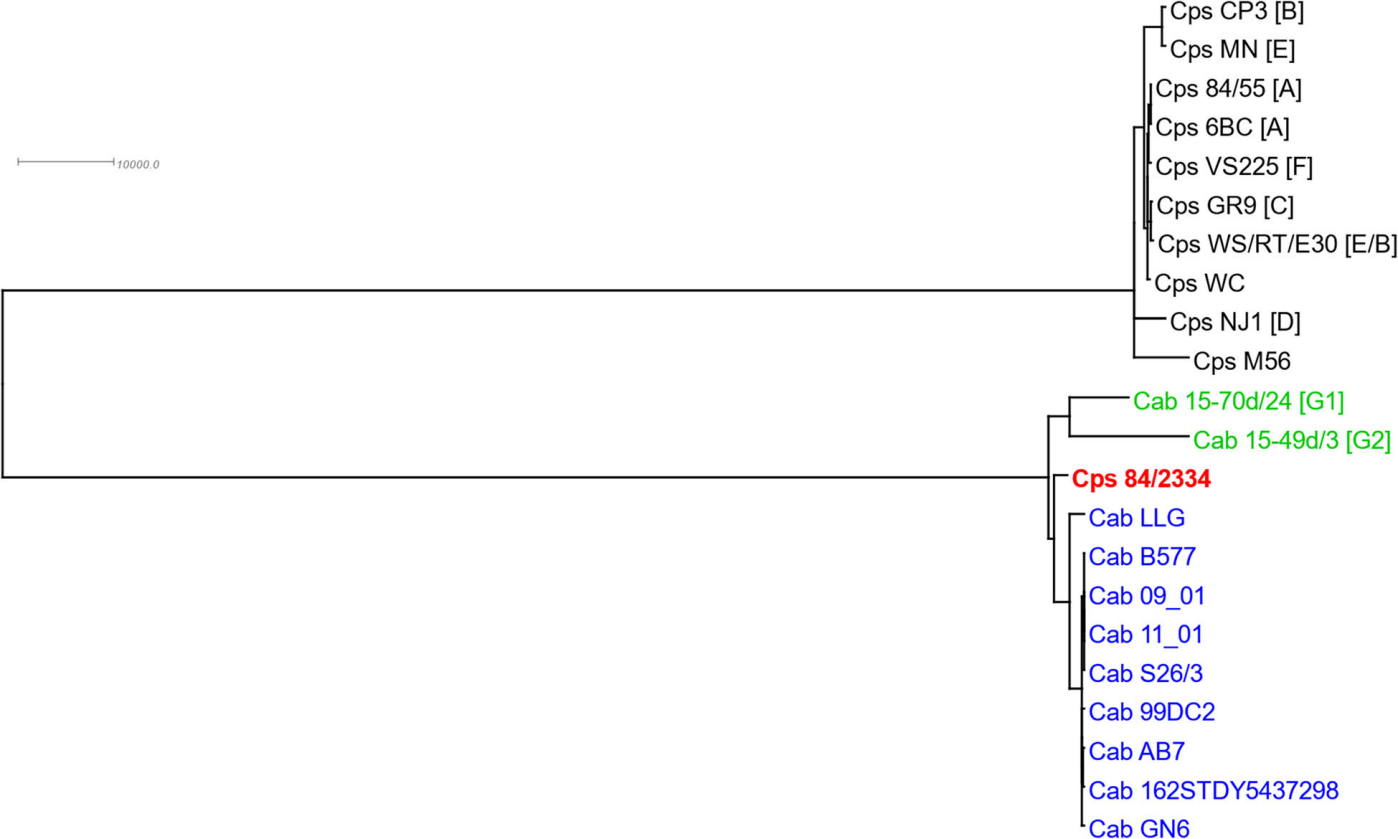
Whole genome phylogenetic analysis informed by recombination. Phylogenetic tree of a whole genome sequence MAFFT alignment of the *C. abortus* (Cab) and *C. psittaci* (Cps) strains shown in Table 2 as derived by Gubbins after removing genomic regions affected by recombination. Genotypes are given in square brackets. FastTree was used as tree builder, and a maximum of 100 iterations was specified in order to guarantee convergence. The tree was midpoint rooted and prepared for publication in Dendroscope. Strain 84/2334 is in bold and red font. Classical and avian *C. abortus* strains are in blue and green fonts, respectively

Whole-genome phylogenetic analysis informed by recombination for the genome (chromosomal sequence) of strain 84/2334 compared to other representative *C. psittaci* and *C. abortus* strains (Table 2) shows a clear separation of the genomes into two clades, with 84/2334 clustering with the *C. abortus* clade (Fig. 4). The two avian *C. abortus* strains 15-59d/3 and 15-70d/24 branch off first from the last common ancestor, with strain 84/2334 branching off second, followed by *C. abortus* variant strain LLG and then all the remaining *C. abortus* strains. Additional analysis, adding an outgroup comprising the genome of *C. pecorum* W73 to the analysis as a control (Additional file 5), conducting the analysis without taking recombination into account (Additional file 6) and NeighborNet analysis (Additional file 7) did not change the structure of the tree and the placement of strain 84/2334 with the *C. abortus* strains. In contrast to the *C. abortus* clade, recombination has a greater effect on the *C. psittaci* clade. For example, while recombination-based analysis identifies strains M56 and NJ1 as the ones branching off first from the *C. psittaci* last common ancestor (Fig. 4), the two strains populate an internal branch of the tree obtained without taking recombination into account (Additional file 6). It should also be noted that whole-genome phylogenetic analysis without taking recombination into account (Additional file 6) and NeighborNet analysis (Additional file 7) clearly place *C. buteonis* strain RSHA between the *C. psittaci* and *C. abortus* clades.

**Fig. 5:**
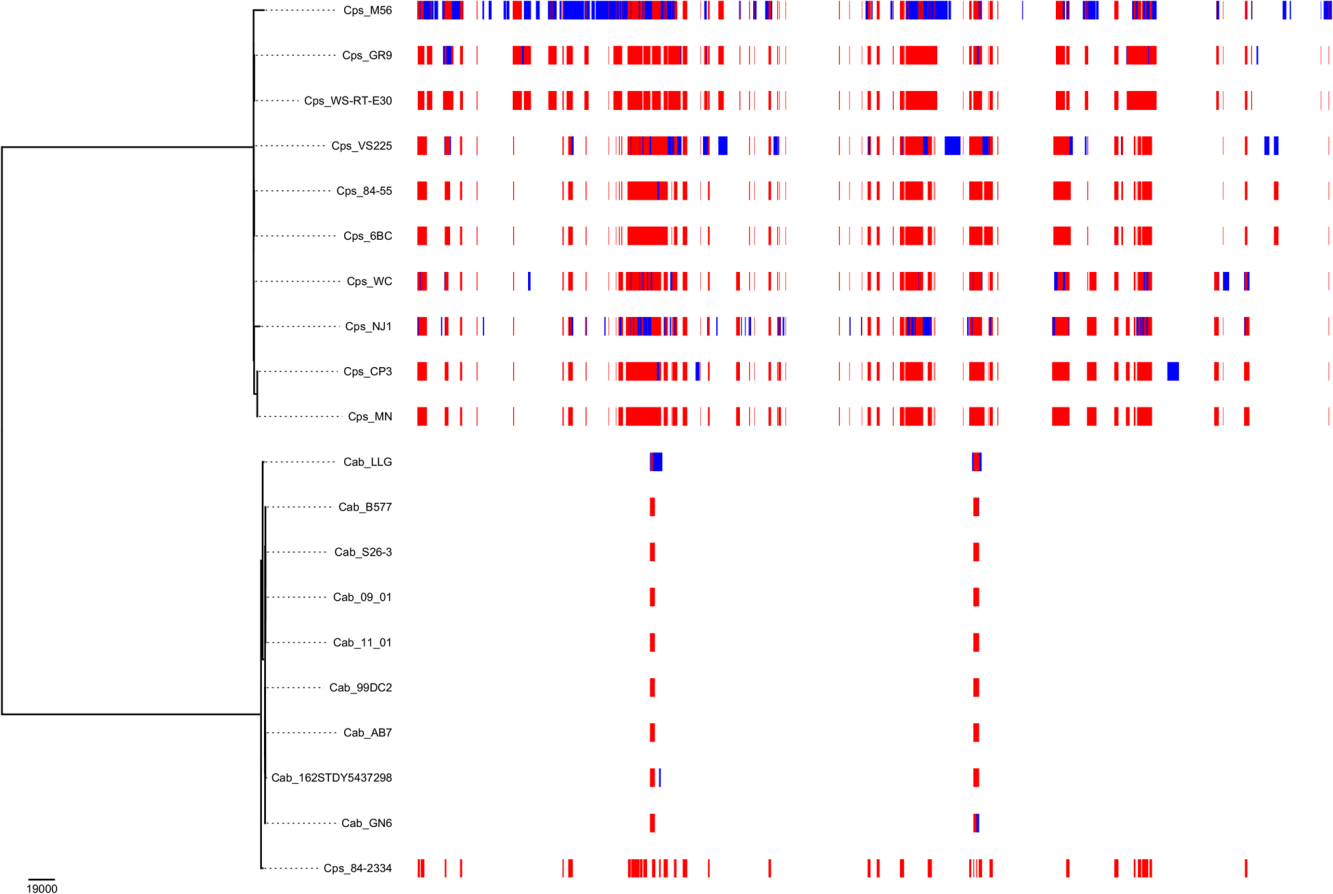
Summary of Gubbins recombination analysis excluding avian *C. abortus* strains 15-59d/3 and 15-70d/24. The red blocks represent recombination events occurring on an internal branch of the phylogenetic tree, which are shared by several strains by common descent. The blue blocks indicate recombination events occurring on terminal branches of the phylogenetic tree, which are unique to a specific strain. The parameters used for the run are those described in Fig. 4

Further details on the regions of recombination identified by Gubbins [41] for all of the strains in Table 1 except the two avian *C. abortus* strains is shown in Fig. 5. The analysis reveals a small number of recombination regions shared within the *C. abortus* clade and a much larger set common to all the *C. psittaci* strains. Strain 84/2334 has an intermediate structure, sharing both the regions shared by *C. abortus* strains and a subset of those common to *C. psittaci*. Notably, several strains (*C. psittaci* M56, VS225, and NJ1; and, to a lesser extent, *C. psittaci* WC and CP3) show greater recombination events unique to the strains. Inclusion of the two avian *C. abortus* strains in the analysis (Additional file 8) reveals additional regions of recombination that are shared by sequences populating the underlying levels of the tree, with 84/2334 exhibiting a pattern almost identical to that of the non-avian *C. abortus* strains.

### Comparative genome analysis and large-scale gene variation in strain 84/2334

Comparative analysis of the genome of strain 84/2334 with each of the genomes of the *C. abortus* and *C. psittaci* strains in Table 2 shows a high level of sequence conservation, specifically in terms of gene content and order (Fig. 2 and Additional file 9). The level of sequence similarity of strain 84/2334 to the other genomes as indicated by blastn matches visualised using CGView (Fig. 2) and the Artemis Comparison Tool (ACT) (Additional file 9) appears greater with *C. psittaci* strains 84/55, 6BC, VS225, CP3, NJ1, MN and WC. The main differences occur in the plasticity zone (PZ), polymorphic membrane protein (Pmp) loci and transmembrane-head protein family (TMH) loci of *C. psittaci* strains GR9, M56 and WS/RT/E30.

#### Plasticity zone

**Fig. 6:**
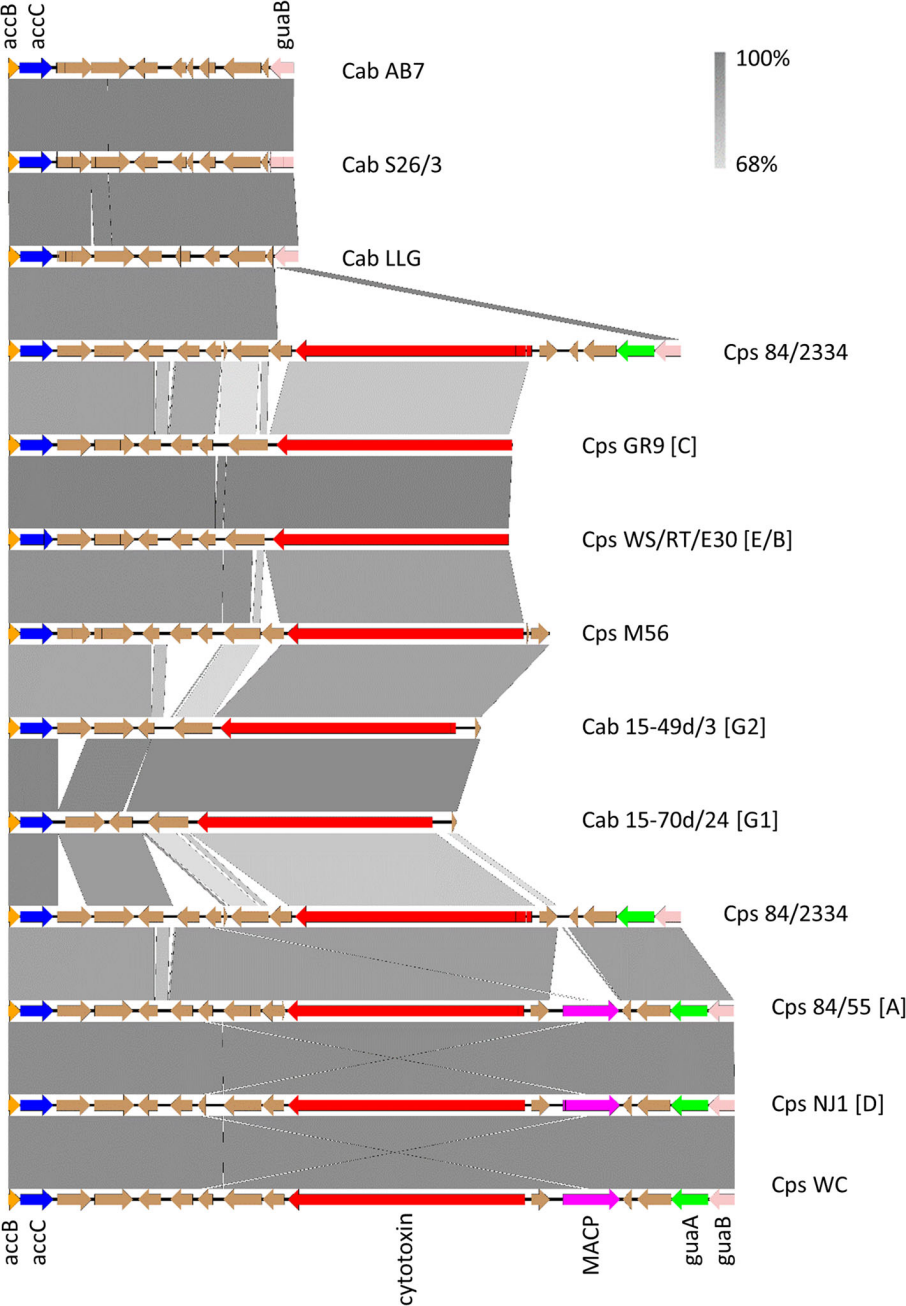
Comparative analysis of the genes present in the plasticity zone of *C. psittaci* strain 84/2334. Comparison of nucleotide matches (computed using blastn) between the genes *acc*B (orange) and *gua*B (pink) in *C. abortus* and *C. psittaci* species and genotypes. *Chlamydia psittaci* genotypes B, E, F (not shown) are identical in gene content to genotypes A, D and WC. The presence of *acc*C (dark blue), *gua*A (green), MACP (purple), cytotoxin (red) and hypothetical protein (brown) genes are as indicated. Vertical lines through the arrows indicate point or frame-shift mutations. The orientation of coding sequences in the forward and reverse frames are indicated by the direction of the block arrows. The level of BLAST identity between the sequences is indicated by the degree of grey shading in the vertical bars. The figure was generated using EasyFig

The PZ of strain 84/2334 most closely resembles that of *C. psittaci* genotypes A, B, D, E, F and WC in terms of gene content, with the one exception, which is that it does not possess the gene encoding the membrane attack complex/perforin domain-containing protein (MACP) (Fig. 6). MACP is also absent in the PZ region of *C. abortus*, avian *C. abortus* and *C. psittaci* genotypes C, E/B and M56. None of the strains, including 84/2334, encode any of the phospholipase D (PLD) protein family genes or any of the genes involved in L-tryptophan biosynthesis found within or external to the PZ region of some other chlamydial species. Strain 84/2334 is clearly different from the classical *C. abortus* strains, having an additional six genes, including a large cytotoxin gene (DZK34_02910) and *guaB* (DZK34_02935) in addition to 4 predicted hypothetical protein genes. However, the large cytotoxin gene is present in the two avian *C. abortus* strains. Phylogenetic analysis of the predicted protein product of the 84/2334 large cytotoxin gene shows that it clusters with the equivalent predicted proteins of the strains belonging to *C. psittaci* genotypes A (6BC and 85/55), B (CP3), D (NJ1), E (MN), F (VS225) and WC, while those of the avian *C. abortus* strains cluster with *C. psittaci* strains C (GR9), E/B (WS/RT/E30) and M56 (Additional file 10). Indeed, the PZ regions of the two avian *C. abortus* strains most closely resemble those of these same *C. psittaci* genotypes (C, E/B, M56), apart from there being fewer of the hypothetical protein genes present between *acc*C and the cytotoxin gene.

#### TMH/Inc. protein family

**Fig. 7:**
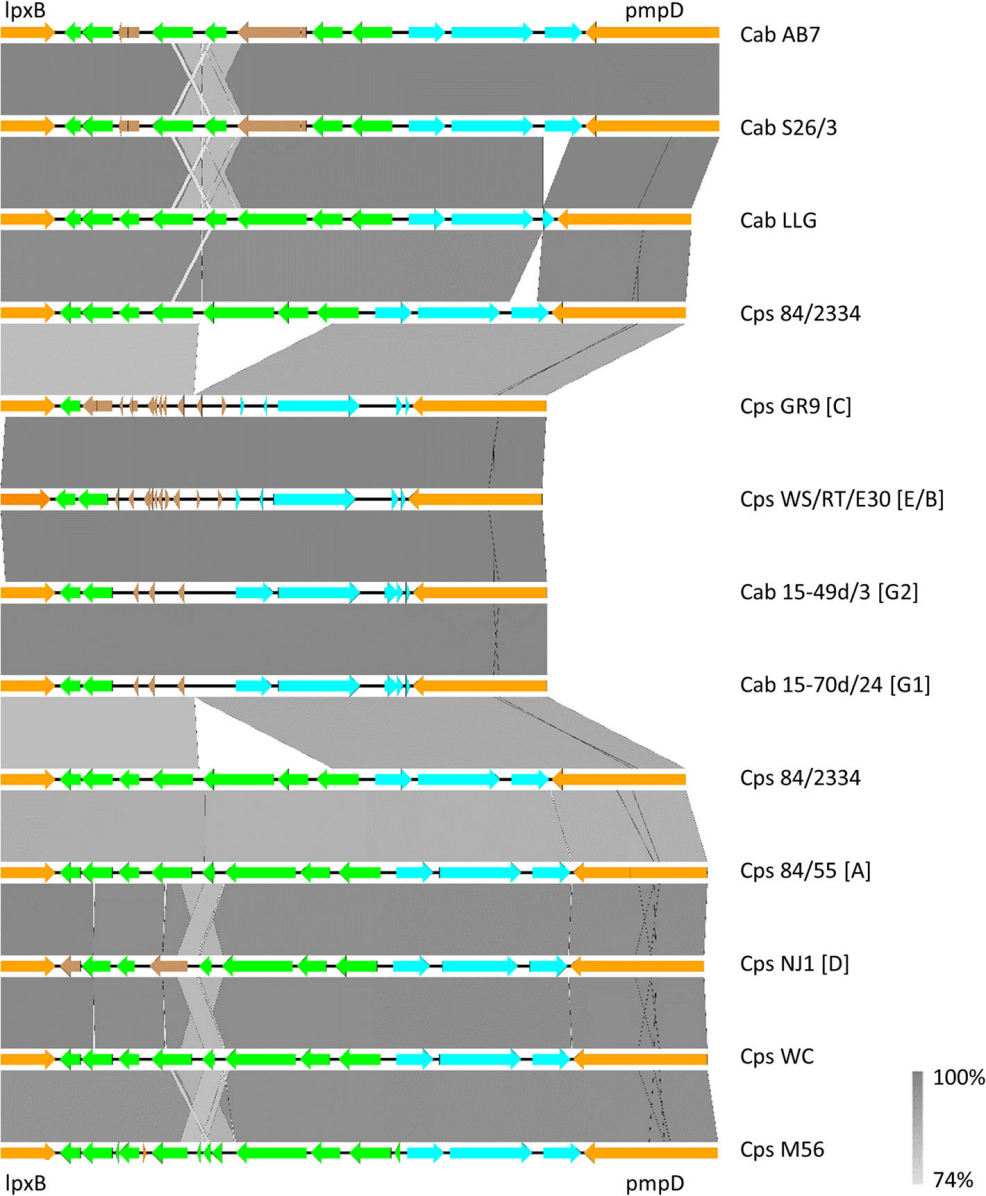
Comparative analysis of the genes present in the transmembrane head of *C. psittaci* strain 84/2334. Comparison of nucleotide matches (computed using blastn) between the genes *lpxB* and *pmpD* (both orange) in *C. abortus* and *C. psittaci* species and genotypes. *Chlamydia psittaci* genotypes B, E, F (not shown) are identical in gene content to genotypes A, D and WC. The presence of TMH/Inc. (green) and hypothetical protein genes/gene remnants (brown) are as indicated. The 3 genes coding for proteins of unknown function are also indicated (blue). Vertical lines through the arrows indicate point or frame-shift mutations. The orientation of coding sequences in the forward and reverse frames are indicated by the direction of the block arrows. The level of BLAST identity between the sequences is indicated by the degree of grey shading in the vertical bars. The figure was generated using EasyFig

A comparison of the TMH loci of *C. abortus* and *C. psittaci* strains shows them to have a high degree of similarity, with the majority of the *C. psittaci* strains (from genotypes A, B, E, F, WC) carrying all 11 of the genes found in *C. abortus*, differing only in pseudogene content (Fig. 7). Strain 84/2334 is also very similar in content to these strains/genotypes other than lacking the CAB766 gene (from *C. abortus* strain S26/3; ortholog CPSIT_0846 in *C. psittaci* 6BC). In contrast, significant differences were noted for *C. psittaci* strains GR9 and WS/RT/E30, which have a smaller TMH locus with considerably fewer intact genes plus many gene remnants (Fig. 7). The two avian *C. abortus* strains (15-49d/3 and 15-70d/24) do not show homology in this region with the typical *C. abortus* strains or indeed strain 84/2334, rather they are most similar to the smaller TMH loci of *C. psittaci* strains GR9 and WS/RT/E30.

#### Polymorphic membrane protein family

**Fig. 8:**
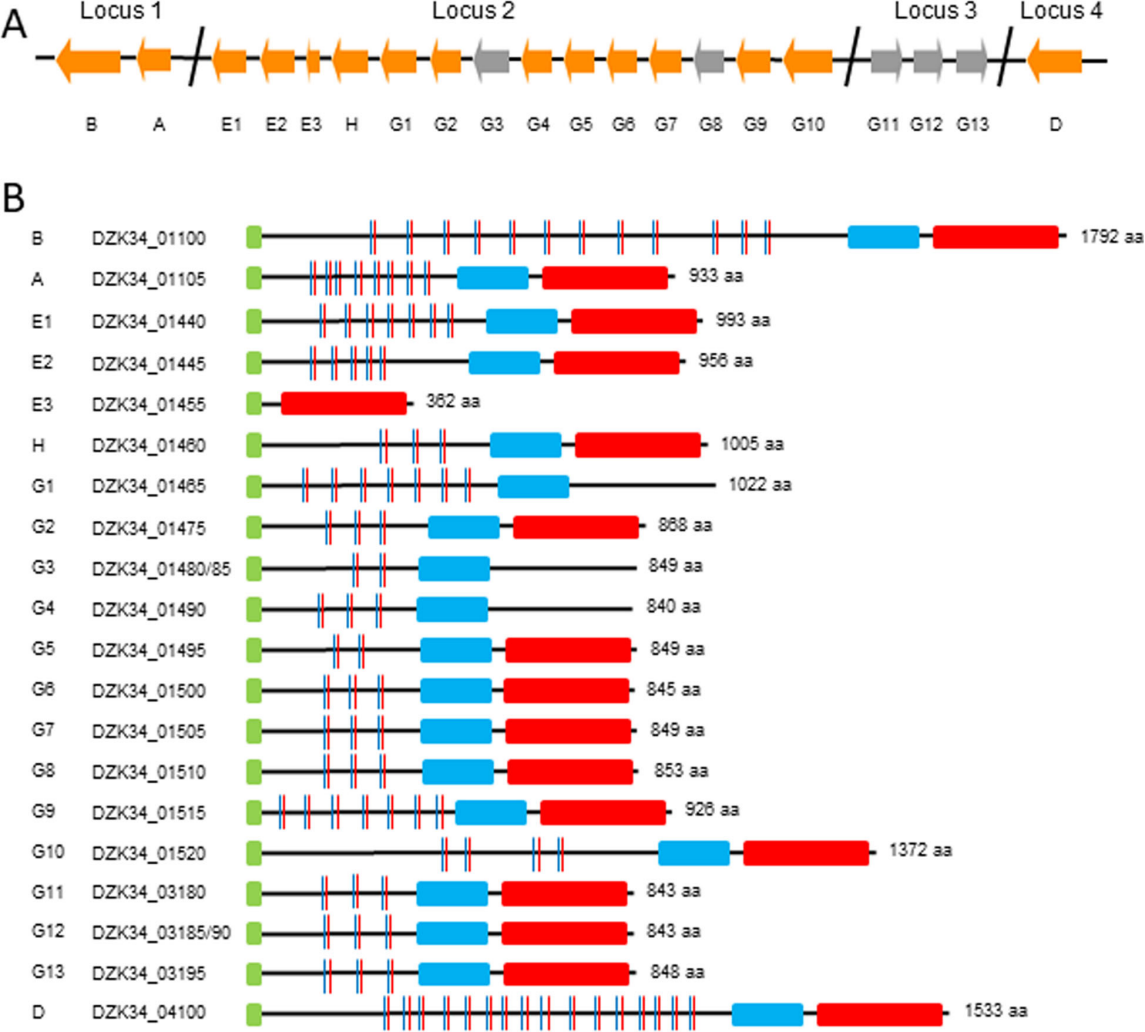
Polymorphic membrane proteins in *C. psittaci* strain 84/2334. **a** Gene families and gene organisation in the four Pmp loci (as indicated) showing intact genes (orange) and pseudogenes (grey), identified following BLAST and phylogenetic comparison with other published Pmps. Pmp gene designations are indicated under each block arrow. **b** Schematic diagram showing the conserved Pmp features, comprising the predicted PmpM domains (blue rectangular block), autotransporter domains (red rectangular block) and pmp passenger domain repeat motifs GG[A/L/V/I][I/L/V/Y] (blue vertical lines) and FXXN (red vertical lines) (Only motifs in which FXXN follows GG[A/L/V/I][I/L/V/Y] are shown). The predicted number of amino acids (aa) is indicated to the right of each gene. Pmp gene identification numbers and gene identification tags (indicated as DZK34_0xxxx) are indicated to the left of each gene

A total of 20 Pmps were identified in strain 84/2334 through comparative comparison with the other strains and through searching for classical Pmp motifs and sequence secondary structure predictions, specifically for a N-terminal domain containing repeat GG[A/L/V/I][I/L/V/Y] and FXXN, a central PmpM domain, a C-terminal autotransporter (AT) domain and a terminal phenylalanine amino acid [42, 43] (Fig. 8). The genes were located in four loci, with single members of the PmpA, PmpB, PmpD and PmpH families and expansion of the PmpE and PmpG families identified, as observed in the other species/strains [42–47]. Following artificial reconstruction of predicted pseudogenes, phylogenetic analysis of the identified sequences confirmed our assignment of the identified Pmps to specific families (Additional file 11). This analysis also showed that strain 84/2334 Pmps from each of the A, B, D, E and H families clustered with the *C. abortus* strains, with the exception of PmpE2 which clustered more closely with the *C. psittaci* clade along with the equivalent proteins of the two avian *C. abortus* strains. Considerable expansion in the PmpG family was observed for strain 84/2334, with a total of 13 Pmps identified (five of which are pseudogenes) (Fig. 8). The two additional Pmps observed in strain 84/2334 (G7 and G12 in Fig. 8) compared to the classical *C. abortus* strains were determined to be gene duplications of two of the PmpG family members that carry homopolymeric (nucleotide ‘G’) tracts. One of these duplications (PmpG12) present in locus 3 (Fig. 8) is a pseudogene, where the other two PmpG proteins (PmpG11 and PmpG13), from which comparative genome analysis suggests it has arisen, are also pseudogenes. The frame-shifts in all three genes occur in homopolymeric (nucleotide ‘G’) tracts. The other gene duplication (PmpG7) in locus 2, as well as PmpG6 (intact gene) and PmpG8 (pseudogene) from which comparative analysis suggests the duplication has arisen, all similarly possess homopolymeric ‘G’ tracts.

## Discussion

In this study, application of a previously developed MLST typing system [32], as well as cluster analysis of MLST allelic profiles/STs, extends previous observations showing clustering of the 84/2334 strain with classical *C. abortus* strains [28] by also showing clustering with the avian *C. abortus* strains (15-70d/24, 15-49d/3 and 15-58d/44). These results were further supported by rMLST, in silico genome-to-genome distance comparisons and 16S rRNA gene phylogenetic analysis, as well as by analysis utilising the recently developed Pillonel typing system [35], all showing similar clustering of the strain with *C. abortus* species, and closer to classical than avian strains. Interestingly, although not a focus of this study, MLST, rRNA, whole genome and NeighborNet phylogenetic analyses all clearly show that *C. buteonis* is phylogenetically positioned between both *C. psittaci* and *C. abortus* species, as previously suggested [7], and although shares similarities with both, appears to cluster more closely with *C. psittaci* and particularly strain M56.

To characterise strain 84/2334 further we sequenced the whole genome, including plasmid sequence, for a more detailed comparison with previously sequenced representative strains of *C. abortus* and *C. psittaci* genotypes [16, 27, 42, 48–50]. While the chromosomal genome characteristics were essentially similar to those of both *C. abortus* and *C. psittaci* species, the GC content was identical to that of *C. abortus* rather than *C. psittaci* species, again suggesting greater similarity with *C. abortus*. However, strain 84/2334 carries an extrachromosomal plasmid, which is more in keeping with *C. psittaci* species. Chlamydial plasmids, which are non-conjugative and non-integrative (with the exception of tetracycline resistance in *C. suis*), have been found in most chlamydial species, including *C. psittaci*, but to date none have been found in classical *C. abortus* species [51, 52]. However, there have been recent reports of strains carrying plasmids that have been classified as avian *C. abortus* strains [18, 49, 50]. Interestingly, the 84/2334 plasmid sequence appears more closely related to these avian *C. abortus* strains than to those of the *C. psittaci* genotypes, perhaps reflecting an ancestral relationship with classical *C. abortus* strains that have lost their plasmid through a process of reductive evolution, where in their specific niche, in a principally mammalian livestock host, the plasmid is not required for tropism and pathogenesis.

The designation of strain 84/2334 is further supported by whole genome phylogenetic analysis informed by recombination using Gubbins [41], which uses SNP density to identify recombination blocks. This analysis places strain 84/2334 firmly within the *C. abortus* clade, in agreement with the typing analyses and also classifies it as an intermediate between the avian and variant/classical *C. abortus* strains, with branch lengths suggesting a closer evolutionary relationship with classical *C. abortus* strains. Overall, strain 84/2334 appears much closer to the *C. abortus* last common ancestor than to the two avian *C. abortus* strains. Interestingly, recombination has the greatest effect on the phylodynamics of the *C. psittaci* clade, whereby the placement of strains within the tree structure significantly differs depending on whether phylogenetics is informed or not by recombination. These findings are consistent with previous studies showing that the level of diversity within classical *C. abortus* strains is low, with only 724 SNPs within the major *C. abortus* clade and 6718 variable sites within the whole phylogeny (including the variant strain LLG/POS clade) [27], while diversity is much greater in *C. psittaci* with 47,710 variable sites [19]. Overall, the greater accuracy of the phylogenetic tree obtained when taking recombination into account was confirmed by whole genome NeighborNet network analysis, which produced results essentially identical to those of Gubbins for both the 84/2334 and the *C. psittaci* strains.

Thus, all the analyses, both with and without taking recombination into account, agree with 84/2334 being a misclassified *C. abortus* strain that branched off from the *C. abortus* last common ancestor at some point between the avian and the classical *C. abortus* strains. This was supported further through a more detailed analysis of the regions of recombination identified by Gubbins, with strain 84/2334 having an intermediate structure, comprising a couple of regions shared by *C. abortus* strains plus a subset of those common to *C. psittaci*, while inclusion of the avian *C. abortus* strains 15-59d/3 and 15-70d/24 added additional regions of recombination, resulting in a distinctively different pattern and again pointing to 84/2334 being closer in evolution to the non-avian *C. abortus* strains.

The genome of strain 84/2334 shares characteristic synteny in terms of gene content and order with the classical *C. abortus*, avian *C. abortus* and *C. psittaci* strains included in this study, as has been observed for other sequenced chlamydial species [42, 43, 53, 54]. However, differences were observed in a number of genes/loci found in regions of extensive variation that have been suggested to be associated with virulence, niche specificity and disease pathogenesis, particularly the PZ, TMH and Pmp loci.

The PZ region, which is defined as bounded by genes inosine-5′-monophosphate dehydrogenase (*guaB*) and acetyl-CoA carboxylase (*accB*), has been found to vary markedly in sequence size (approx. 12–82 kb) and gene content (11–44) across chlamydial species [10, 43]. This highly variable region has been found to carry genes encoding proteins involved in carbohydrate/lipid metabolism (*accBC*), purine metabolism (*guaAB-add*) and tryptophan biosynthesis (*trpABFCDR, kynU, prsA*), as well as genes encoding a membrane attack complex/perforin domain-containing protein (MACP), a phospholipase D (PLD) family of proteins, a varying number of cytotoxin/adherence factor proteins and a number of hypothetical proteins of unknown function. *Chlamydia abortus* has one of the smallest PZ regions amongst chlamydial species, spanning approx. 12 kb, while the PZ of *C. psittaci* spans approx. 21–30 kb depending on genotype. While these regions differ extensively between *C. abortus* and *C. psittaci*, no variation in gene order or content within *C. abortus* species has been observed [27]. In contrast, within *C. psittaci* species there are large differences in gene content at these locations in the different genotypes, perhaps reflecting wide-ranging differences in host specificity. The PZ of strain 84/2334, in keeping with classical *C. abortus*, avian *C. abortus* and *C. psittaci* strains does not contain any of the genes involved in L-tryptophan biosynthesis found within or external to the PZ region of some other chlamydial genomes. Overall, while the PZ of 84/2334 is most similar to that of *C. psittaci* possessing an additional number of similar hypothetical proteins and a cytotoxin gene, it lacks the MACP gene, which is also absent in *C. abortus*. MACP has been suggested to be involved in assisting PLDs in lipid acquisition and processing [55], while no PLD proteins were similarly identified in 84/2334 or indeed any of the strains. Although the function of the cytotoxin is unclear, related cytotoxins in *E. coli* and *C. difficile* have a role in glycosylation of Rho and Ras GTP-binding proteins, inhibiting lymphocyte activation, host signalling and blocking the induction of interferon-gamma (IFN-γ) [56, 57]. As IFN-γ decreases the availability of L-tryptophan and can lead to resolution of chlamydial infections [58, 59], the ability to block its production may be an important virulence determinant that allows the organism to form persistent subclinical infections, particularly when these pathogens appear to lack the ability to synthesise tryptophan with the absence of the tryptophan biosynthesis operon. However, it is unclear whether the encoded cytotoxin in strain 84/2334 is actively expressed or truncated at the amino terminus as a number of frameshift mutations are present at the 5′ end of the gene. But this gene is present in all of the *C. psittaci* strains/genotypes, including the avian *C. abortus* strains, although phylogenetic analysis shows that it clusters with different *C. psittaci* genotypes than the avian *C. abortus* strains, perhaps reflecting a different evolutionary path and the ultimate loss of this gene in classical *C. abortus* strains.

Another region that has been identified as important in terms of virulence is the TMH locus that typically has 11 genes between the lipid A disaccharide synthase gene (*lpxB*) and the polymorphic membrane protein D (*pmpD*) [42] that encode proteins with either paired (*n* = 8) or single (*n* = 3) N-terminal transmembrane domains followed by alpha-helical coiled-coil domains of varying lengths, with an amino acid composition rich in leucine, glutamate and serine residues or contain conserved domains of unknown function [42, 48]. These proteins, which lack a signal sequence, are thought to be secreted via the Type III Secretion System and have been associated with the chlamydial inclusion membrane, chlamydial growth and host inflammatory responses [60–62]. They are also thought to be related to the Inc-protein family, which similarly possess no primary sequence similarity but have unique paired hydrophobic domains in either the N-terminal (IncA) or C-terminal (IncB and IncC) regions (all three Inc. proteins are present in strain 84/2334 and all the other *C. abortus* and *C. psittaci* strains included in this study) [27, 44, 48]. Although there are few differences in gene content between strain 84/2334 and *C. abortus* and most *C. psittaci* genotypes, with some differences in pseudogene content, the main difference is in the absence of the CAB766 gene in 84/2334. This gene encodes the only TMH protein, termed Inc766, to be characterised from this region to date, and which has been shown to form oligomers and to be localised to the extra-inclusion space [63]. This possibly suggests a role for this protein and other TMH proteins in vesicular trafficking and modulation of host cell functions, as suggested for other Incs [60, 63, 64]. In contrast, the TMH region for the two avian *C. abortus* strains and *C. psittaci* strains GR9 and WS/RT/E30 is much smaller with considerably fewer intact genes plus many gene remnants, again perhaps reflecting a different evolutionary process for these avian strains compared to strain 84/2334.

A major source of large-scale variation among *Chlamydia* spp. is in the Pmp proteins, which are members of the type V “autotransporter (AT)” secretion system [65]. Although the function of these proteins has largely to be determined they have been suggested to be involved in niche adaptation and host immune evasion, with specific functions on host cell adherence, molecular transport and cell wall associated functions [66–68]. The number of Pmps in the family *Chlamydiaceae* has been shown to vary considerably from 9 to 21 depending on species, with *C. abortus* having 18 and *C. psittaci* 17–21 [42, 44–47, 53, 69]. These Pmps are broadly classified into six families (A, B/C, D, E/F, G/I, and H) that share little primary sequence homology but which have distinct characteristic features, possessing an N-terminal passenger domain with a variable number of repeat motifs GG[A/L/V/I][I/L/V/Y] and FXXN, a central PmpM domain, a C-terminal AT domain and all end with the amino acid phenylalanine. In contrast, Pmps within a family share primary sequence homology both within and across species/strains. In this study we identified a total of 20 Pmps in strain 84/2334, with phylogenetic analysis showing that individual family members cluster more closely with the equivalent proteins from *C. abortus*, suggesting a closer evolutionary relationship. The additional two Pmps in 84/2334 to those found in *C. abortus* strains result from gene duplication and expansion of the G family from 11 to 13, similar to the number observed in *C. psittaci* (*n* = 10–14). However, these gene duplications and the genes they have arisen from possess homopolymeric tracts that are thought to be subject to phase variation by slip-strand mispairing [42]. These observations, coupled with the expansion and variation observed in this PmpG family across the *C. abortus* and *C. psittaci* species/strains, highlights that this is the most diverse and rapidly evolving of all the Pmp families, likely arising and evolving as a result of gene duplications and losses in the various chlamydial ancestries and indicative of the observed recombinogenic nature of this region of the genome [27, 42].

## Conclusion

In this study we have fully characterised the *C. psittaci* strain 84/2334 at the whole genome level using existing typing systems, as well as through whole genome sequencing, comparative genomics and recombination analysis. These analyses show an evolutionary relationship between the strain with both *C. abortus* and *C. psittaci* species, in agreement with a previous publication suggesting the strain to be a missing link between *C. psittaci* and *C. abortus* and classifying it as an intermediary strain [21]. However, this study shows that the strain is not intermediary at all, but rather much closer in evolution to the classical non-avian *C. abortus* strains than to the avian *C. abortus* strains or *C. psittaci* species, branching off from the last common ancestor of *C. abortus* between the avian and classical strains. These results coupled with the presence of the plasmid sequence which clusters more closely with the avian *C. abortus* plasmids than the *C. psittaci* or other chlamydial plasmids, along with taking into account similarities and differences in gene content in key regions and loci associated with virulence, niche specificity and pathogenesis are all consistent with strain 84/2334 being an ancestral *C. abortus* species. Therefore, overall, the results of this study support the reclassification of this strain as *C. abortus* species. Future studies should aim to characterise the strain further, investigating its pathogenesis in challenge model systems, as well as with the recent advances in plasmid-based transformation of chlamydial species [70, 71] begin to understand gene function as well as look towards the future development of new novel vaccines.

## Methods

### Cell culture, DNA extraction and genome sequencing

*Chlamydia psittaci* strain 84/2334, isolated from the lungs of an imported yellow-headed Amazon parrot in Germany [31], was grown in Buffalo Green Monkey kidney (BGM; obtained from ECACC General Collection, product number 90092601) cells using standard techniques [72]. Infected cells were harvested to purify chlamydial elementary bodies, as previously published [73], and DNA extracted using a DNeasy Blood & Tissue kit (Qiagen Ltd., Belgium). Genomic DNA was fractionated into smaller fragments (300–800 bp), blunt-ended, adapters ligated onto fragments and attached to DNA-capture beads to generate a single-stranded template library for pyrosequencing on a 454 GS-FLX Titanium (Roche) pyrosequencer, according to the manufacturer’s instructions.

### Mapping, assembly, annotation and comparative analysis

Following quality filtering, de novo assembly and reference mapping assembly to each of the *C. psittaci* genomes in Table 1 was performed using the software program Newbler (version 2.3; 454 Life Sciences, Branford, CT, USA). De novo assembly produced a single contig, with 273x sequencing coverage. Genome annotation was performed using the NCBI prokaryotic genome annotation pipeline (PGAP) [74] and manually curated using Artemis [75]. Pseudogenes were defined as having one or more mutations that would ablate expression (i.e. indel or substitution causing frameshift or stop codon). The origin of replication was determined using Ori-Finder [76] and the genome adjusted so that the first base was upstream of *hemB* in the *oriC* region. To identify any reads mapping to a plasmid, the plasmids of *C. psittaci* strains 6BC and 84/55 (Table 2) were used as reference sequences.

Comparative genome and plasmid analyses of 84/2334 was performed against representative strains of each *C. psittaci* genotype (6BC, 84/55, CP3, GR9, NJ1, MN, VS225, M56, WC [16]), *C. abortus* species (S26/3 [42], AB7 [77], LLG [27, 48]) and avian *C. abortus* strains (genotypes G1 and G2 [18, 50]). For the plasmid comparisons, sequences were oriented to start at a position relative to nucleotide 6848 in strain 84/2334. Global genome and plasmid comparisons were visualised using the Artemis Comparison Tool (ACT) [78] with crunch input files generated by running pairwise blastn comparisons of the sequences using Megablast within the ncbi-blast-2.9.0+ command line software [79]. Genome and plasmid maps were generated using the CGView server [80].

### Molecular genotyping schemes

MLST genotyping of gene fragments of seven housekeeping genes (*enoA, fumC, gatA, gidA, hemN, hlfX and oppA*) of *C. psittaci* strain 84/2334 and representative strains from *Chlamydiaceae* species, was performed as previously described [28, 32]. Genes were concatenated, aligned using MAFFT and a phylogenetic tree estimated in TOPALi as detailed in the following section (Phylogenetic and network analysis). Cluster analysis based on defined allelic profiles or sequence types (ST) for each individual isolate, as well as STs from other representative isolates for all *Chlamydiaceae* species (except *C. trachomatis*) in the online database [36, 81] was conducted to generate minimum spanning trees that were visualised using GrapeTree [82]. Ribosomal MLST analysis to determine species classification was conducted using the *rps* gene database [34, 83]. Digital DNA-DNA hybridization to determine species was conducted using the Genome-to-Genome Distance Calculator 2.1 [33, 84].

A classification scheme, based on five discriminant proteins (Adk, FtsK, HemL, PepF and RpoN) for species designation, was applied to strain 84/2334, as previously described [35]*. *Protein sequences for all strains were identified by BLAST analysis. Sequence distances (% ID) for each of the five proteins from strain 84/2334 with each of the equivalent proteins from the* C. psittaci *and *C. abortus *strains and genotypes in Table 2 were calculated by aligning sequences in MegAlign 15 (Lasergene software, DNASTAR Inc., Madison, WI, USA) Clustal Omega [85]. Proteins with a %ID ≥95% for Adk and HemL, ≥ 96% for PepF and RpoN, and ≥ 98% for FtsK, indicate they are classified as the same species.

### Phylogenetic and network analysis

Whole genome, plasmid, gene (16S rRNA, concatenated MLST housekeeping genes) and protein (Pmps, cytotoxins) sequences were aligned using MAFFT v7.450 [86] or Clustal Omega. Phylogenetic trees were estimated in IQ-Tree v2.0.5 [87] or TOPALi v2.5 [88] by Maximum Likelihood using GTR + G + I (concatenated MLST housekeeping genes), TVM + F + R6 (genomes), TIM + G (plasmids), JTTDCMut + F + I + G4 (Cytotoxins; PmpsABDH), JTT + F + R3 (PmpsE) and JTT + F + R5 (PmpsG) substitution and rate models or by NeighbourJoining using F84 + G (16S rRNA genes) substitution and rate models. Models were selected according to Bayesian information criterion (BIC). Bootstrap analyses were performed on 1000 replicate trees. All trees were midpoint rooted, unless otherwise indicated in the figure legend. Trees were prepared for publication using Dendroscope 3 [89]. Phylogenetic network analysis for inferring evolutionary relationships between the MAFFT aligned genome species and strains was performed using SplitsTree v4.15.1 [90].

### Recombination analysis

To investigate the effect of recombination on the phylogeny Gubbins version 2.4.1 [41] was used. Genome sequences (Table 2) were aligned using MAFFT v7.471 [86]. Recombination analysis was performed with and without the two avian *C. abortus* strains (15-70d/24 and 15-49d/3) present in the alignments.

### Plasticity zone, transmembrane head and polymorphic membrane protein gene analysis

The PZ region, spanning genes between *gua*B and *acc*B, and TMH/Inc. locus, typically encoding genes between *lpx*B and *pmp*D *w*ere manually identified in each of the *C. abortus* and *C. psittaci* genomes using ACT [78]. BLAST searches were also performed to confirm gene content within these loci. PZ and TMH/Inc. loci were extracted for each of the genomes and linear comparisons were produced using Easyfig for Windows version 2.2.5 [91].

Gene sequences for the polymorphic membrane proteins (Pmps) were manually checked in each of the *C. abortus* and *C. psittaci* genomes using ACT [78] to determine functionality and identify pseudogenes i.e. where there were one or more mutations that would ablate expression. For the purposes of this study all identified pseudogenes were artificially constructed in silico by comparison with intact homologous genes in other strains/species so that they could be included in the analysis. Prior to the phylogenetic analysis of Pmp gene families detailed in the previous section (Phylogenetic and network analysis), the Pmp protein sequences were initially classified into their respective family groups (A, B, D, E, G and H) through phylogenetic analysis of a single alignment of all the sequences in IQ-Tree. Pmp-specific C-terminal autotransporter β-barrel domains and the conserved PmpM middle domain motifs were identified using the Pfam HMM database [92]. Predicted Pmp passenger domain repeat motifs were identified manually where GG[A/L/V/I][I/L/V/Y] motifs were closely followed by an FXXN motif.

## Supplementary Information

**Additional file 1: Table S1.** Results of rMLST analysis to determine species classification using the *rps* gene database at https://pubmlst.org/species-id.

**Additional file 2: Fig. S1.** Minimum spanning tree illustrating cluster analysis of MLST sequence type (ST) profiles. Cluster analysis is based on nucleotide differences in seven MLST housekeeping gene fragments *(enoA*, *fumC*, *gatA*, *gidA*, *hemN*, *hlfX* and *oppA*). STs for strain *C. psittaci* strain 84/2334 and representative strains from *Chlamydiaceae* species (excluding *C. trachomatis*) are indicated (genotypes are given in square brackets within tree; numbers in square brackets in key indicate total strains included for each species).

**Additional file 3: Fig. S2.** Phylogenetic tree of a 16S rRNA gene alignment of strain 84/2334 and other *Chlamydiaceae* species. The consensus tree for the 1470 bp alignment was estimated in TOPALi by Neighbour Joining using a F84 + G substitution and rate heterogeneity model and 100 non-parametric bootstrap replicates. The tree is midpoint rooted and bootstrap support is indicated by the number at the node. The scale bar indicates the expected substitutions per site. Genotypes are given in square brackets. The tree was prepared in Dendroscope. Strain 84/2334 is in bold and red font.

**Additional file 4: Table S2.** Results of digital DNA-DNA hybridization to determine species using the Genome-to-Genome Distance Calculator 2.1 at http://ggdc.dsmz.de/ggdc.php.

**Additional file 5: Fig. S3.** Whole genome phylogenetic analysis informed by recombination with added outgroup. Phylogenetic tree of a whole genome sequence MAFFT alignment of the *C. abortus* (Cab) and *C. psittaci* (Cps) strains shown in Table 2 as derived by Gubbins after removing genomic regions affected by recombination. Genotypes are given in square brackets. The sequence of *C. pecorum* W73 was additionally provided as outgroup. FastTree was used as tree builder, and a maximum of 100 iterations was specified in order to guarantee convergence. Tree was midpoint rooted and prepared using Dendroscope. Strain 84/2334 is in bold and red font. Classical and avian *C. abortus* strains are in blue and green fonts, respectively.

**Additional file 6: Fig. S4.** Whole genome phylogenetic analysis. Phylogenetic tree of a whole genome sequence MAFFT alignment of the *C. abortus* (Cab) and *C. psittaci* (Cps) strains shown in Table 2, and *C. buteonis* strain RSHA. The consensus tree was estimated in IQ-Tree by Maximum Likelihood using a TVM + F + R2 substitution and rate heterogeneity model, according to BIC model selection, and 100 non-parametric bootstrap replicates. The tree is midpoint rooted and bootstrap support is indicated by the number at the node. The scale bar indicates the expected substitutions per site. Genotypes are given in square brackets. The tree was prepared in Dendroscope. Strain 84/2334 is in bold and red font. Classical and avian *C. abortus* strains are in blue and green fonts, respectively.

**Additional file 7: Fig. S5.** Whole genome NeighborNet network analysis. Phylogenetic network of a whole genome sequence alignment of the *C. abortus* (Cab) and *C. psittaci* (Cps) strains shown in Table 2, using the NeighborNet distances transformation (Ordinary Least Squares variance and Lambda Frac of 1.0) and EqualAngle splits transformation. The scale bar indicates the expected substitutions per site. Genotypes of *C. psittaci* and avian *C. abortus* strains are indicated in brackets. The figure was generated using SplitsTree4. Strain 84/2334 is in bold and red font.

**Additional file 8: Fig. S6.** Summary of Gubbins recombination analysis including avian *C. abortus* strains 15-59d/3 and 15-70d/24. The red blocks represent recombination events occurring on an internal branch of the phylogenetic tree, which are shared by several strains by common descent. The blue blocks indicate recombination events occurring on terminal branches of the phylogenetic tree, which are unique to a specific strain. The parameters used for the run are those described in Fig. 4.

**Additional file 9: Fig. S7.** Comparative genome analysis of *C. psittaci* 84/2334. Whole genome comparisons of *C. psittaci* strain 84/2334 and (A) *C. abortus* strains S26/3, AB7 and LLG and (B) representative *C. psittaci* strains VS225, 84/55, GR9 and M56 depicting amino acid matches computed using Megablast blastn. Homology matches are indicated by the red vertical bars, while inverted matches (indicating areas of recombination) are coloured blue. Horizontal grey bars represent the forward and reverse strands of DNA with CDSs marked as arrows. The main regions of difference occur in two of the Pmp loci (circled areas 1 and 3), the PZ region (circled area 2) and TMH loci (circled area 4). Please note that the gaps shown for *C. abortus* strain LLG compared to 84/2334 in Pmp loci (circled areas 1 and 3) are due to the sequences not being complete in these regions rather than homology differences. The figure was generated using the Artemis Comparison Tool (ACT).

**Additional file 10: Fig. S8**. Phylogenetic analysis of chlamydial cytotoxin predicted protein sequences. The consensus tree was estimated in IQ-Tree by Maximum Likelihood using a JTTDCMut + F + I + G4 substitution and rate heterogeneity model, according to BIC model selection, and 100 non-parametric bootstrap replicates. The tree is midpoint rooted and bootstrap support is indicated by the number at the node. The scale bar indicates the expected substitutions per site. The tree was prepared in Dendroscope. Strain 84/2334 is in bold and red font.

**Additional file 11: Fig. S9**. Polymorphic membrane protein family phylogenies. Consensus trees for singleton PmpA, PmpB, PmpD and PmpH family members (A), for the PmpE family members (B) and PmpG family members (C) for strain 84/2334 and the *C. abortus* and *C. psittaci* strains/genotypes shown in Table 2 were estimated in IQ-Tree by Maximum Likelihood using substitution and rate heterogeneity models JTTDCMut + F + I + G4, JTT + F + R3 and JTT + F + R5, respectively, according to BIC model selection, and 100 non-parametric bootstrap replicates. The trees were midpoint rooted and bootstrap support is indicated by the number at the node (only values greater than 70 are shown). The scale bar indicates the expected substitutions per site. The trees were prepared in Dendroscope. Strain 84/2334 is in bold and red font. Classical and avian *C. abortus* strains are in blue and green fonts, respectively.

## Data Availability

The datasets supporting the conclusions of this article are included within the article (and its additional files) and in the NCBI repository under BioProject accession PRJNA485898. The complete assemblies of both the chromosomal and plasmid sequences are available under accession numbers CP031646 and CP031647, respectively. The dataset(s) supporting the conclusions of this article is (are) available in the [repository name] repository, [unique persistent identifier and hyperlink to dataset(s) in http:// format].

## Abbreviations

ACT: Artemis comparison tool
AT: Autotransporter
EAE: Enzootic abortion of ewes
IFN-γ: Interferon-gamma
MACP: Membrane attack complex protein
MLST: Multi-locus sequence typing
OEA: Ovine enzootic abortion
PLD: Phospholipase D
Pmp: Polymorphic membrane protein
PZ: Plasticity zone
rMLST: Ribosomal multi-locus sequence typing
SNP: Single nucleotide polymorphism
TMH: Transmembrane head
