# Supplementary figures and images for: Whole genome de novo sequencing and comparative genomic analyses suggests that Chlamydia psittaci strain 84/2334 should be reclassified as Chlamydia abortus species
Source: BMC Genomics. 2021 Mar 6;22:159. doi: 10.1186/s12864-021-07477-6 (PMC7937271; doi:10.1186/s12864-021-07477-6)

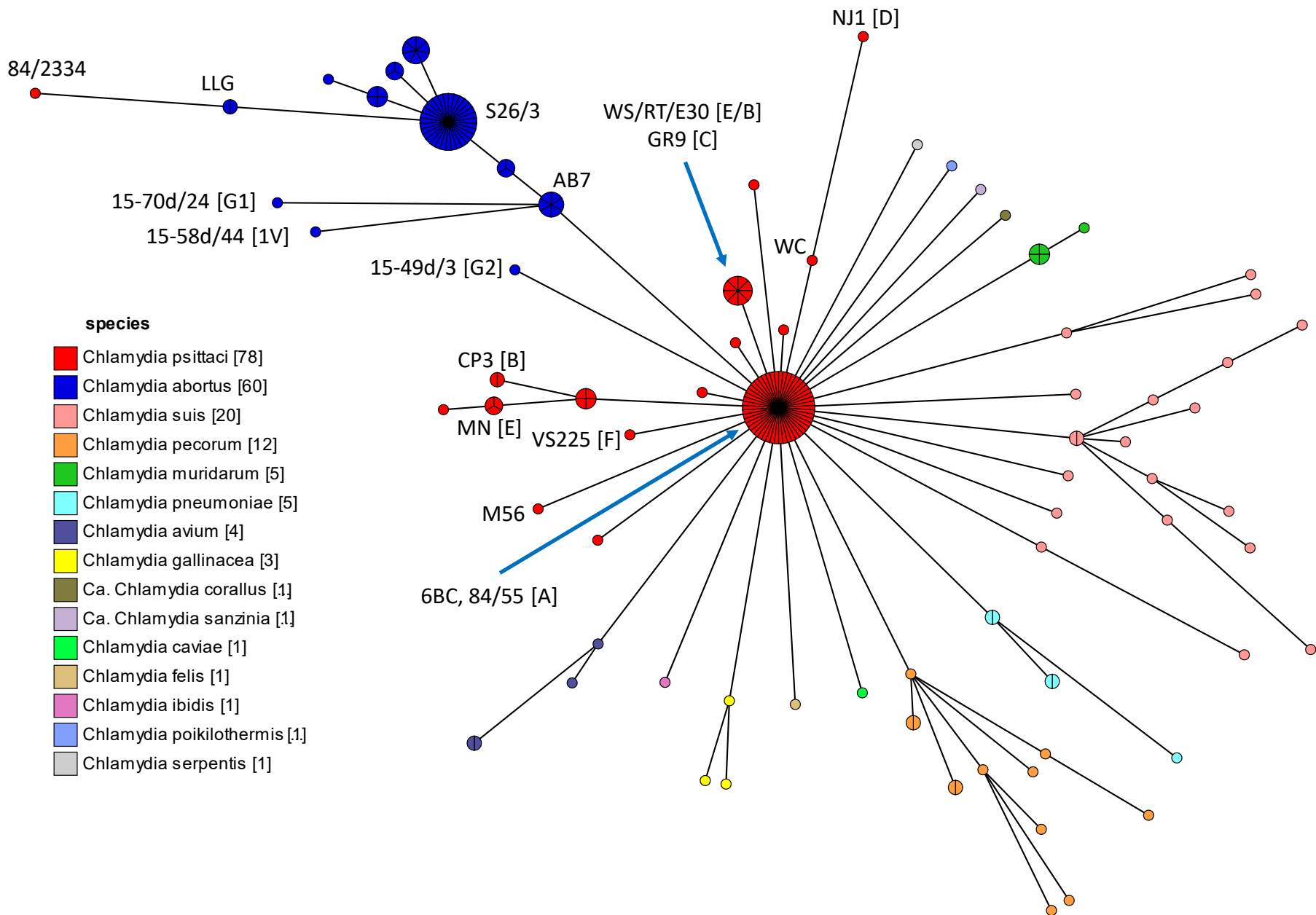

Supplement: Supplementary file 2 — Additional file 2: Fig. S1. Minimum spanning tree illustrating cluster analysis of MLST sequence type (ST) profiles. Cluster analysis is based on nucleotide differences in seven MLST housekeeping gene fragments (enoA, fumC, gatA, gidA, hemN, hlfX and oppA). STs for strain C. psittaci strain 84/2334 and representative strains from Chlamydiaceae species (excluding C. trachomatis) are indicated (genotypes are given in square brackets within tree; numbers in square brackets in key indicate total strains included for each species). [file 12864_2021_7477_MOESM2_ESM.pdf]

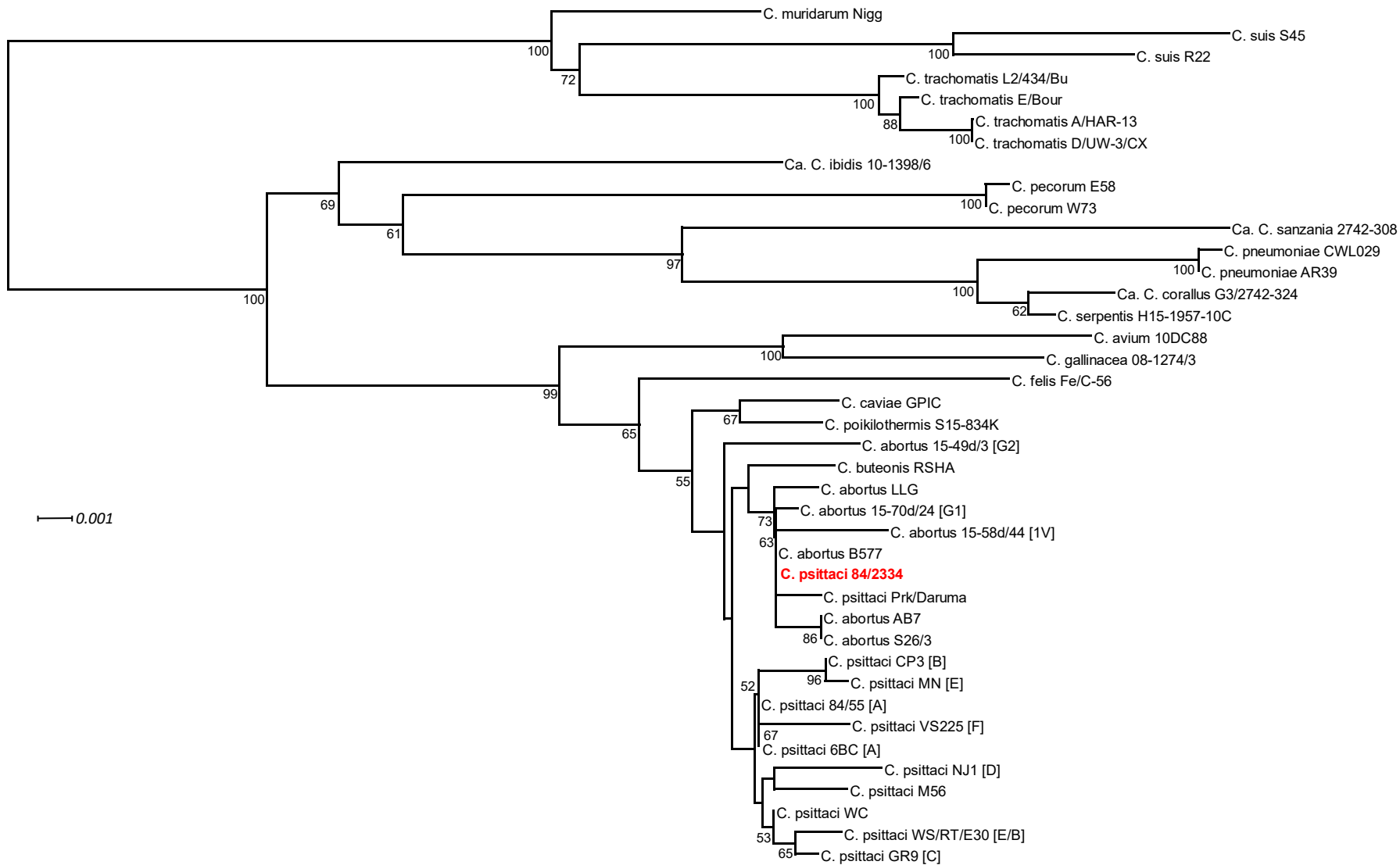

Supplement: Supplementary file 3 — Additional file 3: Fig. S2. Phylogenetic tree of a 16S rRNA gene alignment of strain 84/2334 and other Chlamydiaceae species. The consensus tree for the 1470 bp alignment was estimated in TOPALi by Neighbour Joining using a F84 + G substitution and rate heterogeneity model and 100 non-parametric bootstrap replicates. The tree is midpoint rooted and bootstrap support is indicated by the number at the node. The scale bar indicates the expected substitutions per site. Genotypes are given in square brackets. The tree was prepared in Dendroscope. Strain 84/2334 is in bold and red font. [file 12864_2021_7477_MOESM3_ESM.pdf]

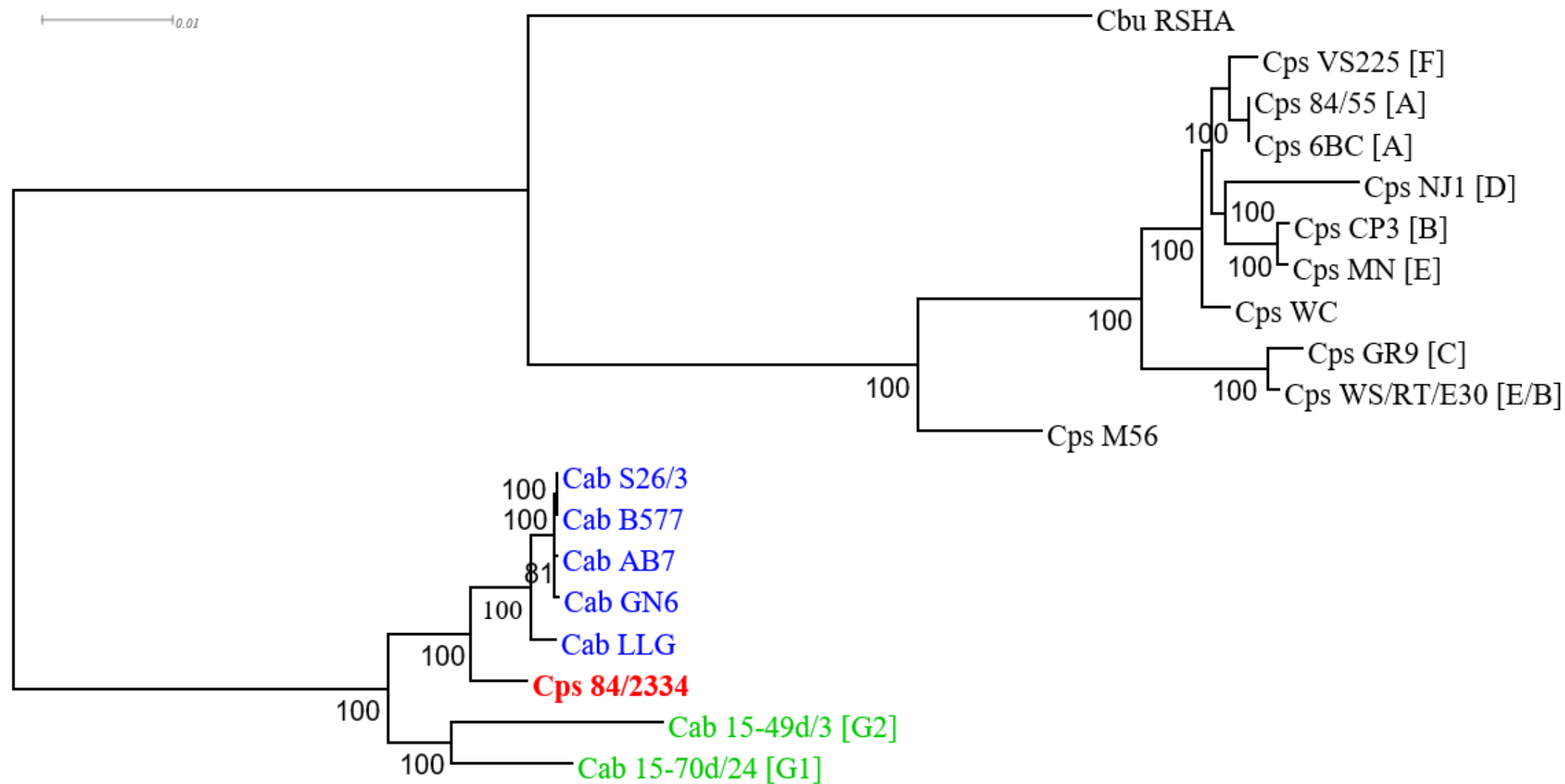

Supplement: Supplementary file 6 — Additional file 6: Fig. S4. Whole genome phylogenetic analysis. Phylogenetic tree of a whole genome sequence MAFFT alignment of the C. abortus (Cab) and C. psittaci (Cps) strains shown in Table 2, and C. buteonis strain RSHA. The consensus tree was estimated in IQ-Tree by Maximum Likelihood using a TVM + F + R2 substitution and rate heterogeneity model, according to BIC model selection, and 100 non-parametric bootstrap replicates. The tree is midpoint rooted and bootstrap support is indicated by the number at the node. The scale bar indicates the expected substitutions per site. Genotypes are given in square brackets. The tree was prepared in Dendroscope. Strain 84/2334 is in bold and red font. Classical and avian C. abortus strains are in blue and green fonts, respectively. [file 12864_2021_7477_MOESM6_ESM.pdf]

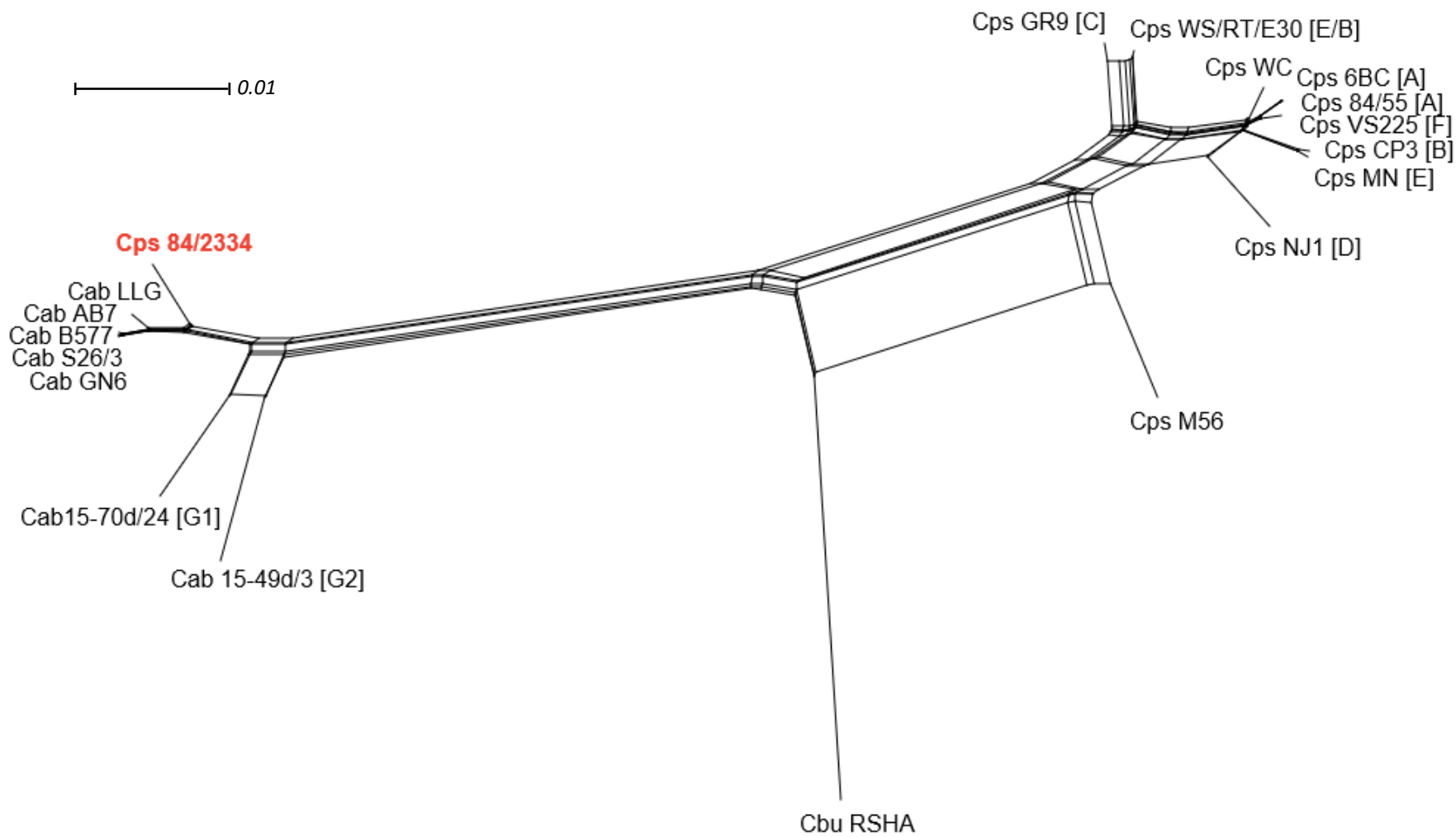

Supplement: Supplementary file 7 — Additional file 7: Fig. S5. Whole genome NeighborNet network analysis. Phylogenetic network of a whole genome sequence alignment of the C. abortus (Cab) and C. psittaci (Cps) strains shown in Table 2, using the NeighborNet distances transformation (Ordinary Least Squares variance and Lambda Frac of 1.0) and EqualAngle splits transformation. The scale bar indicates the expected substitutions per site. Genotypes of C. psittaci and avian C. abortus strains are indicated in brackets. The figure was generated using SplitsTree4. Strain 84/2334 is in bold and red font. [file 12864_2021_7477_MOESM7_ESM.pdf]

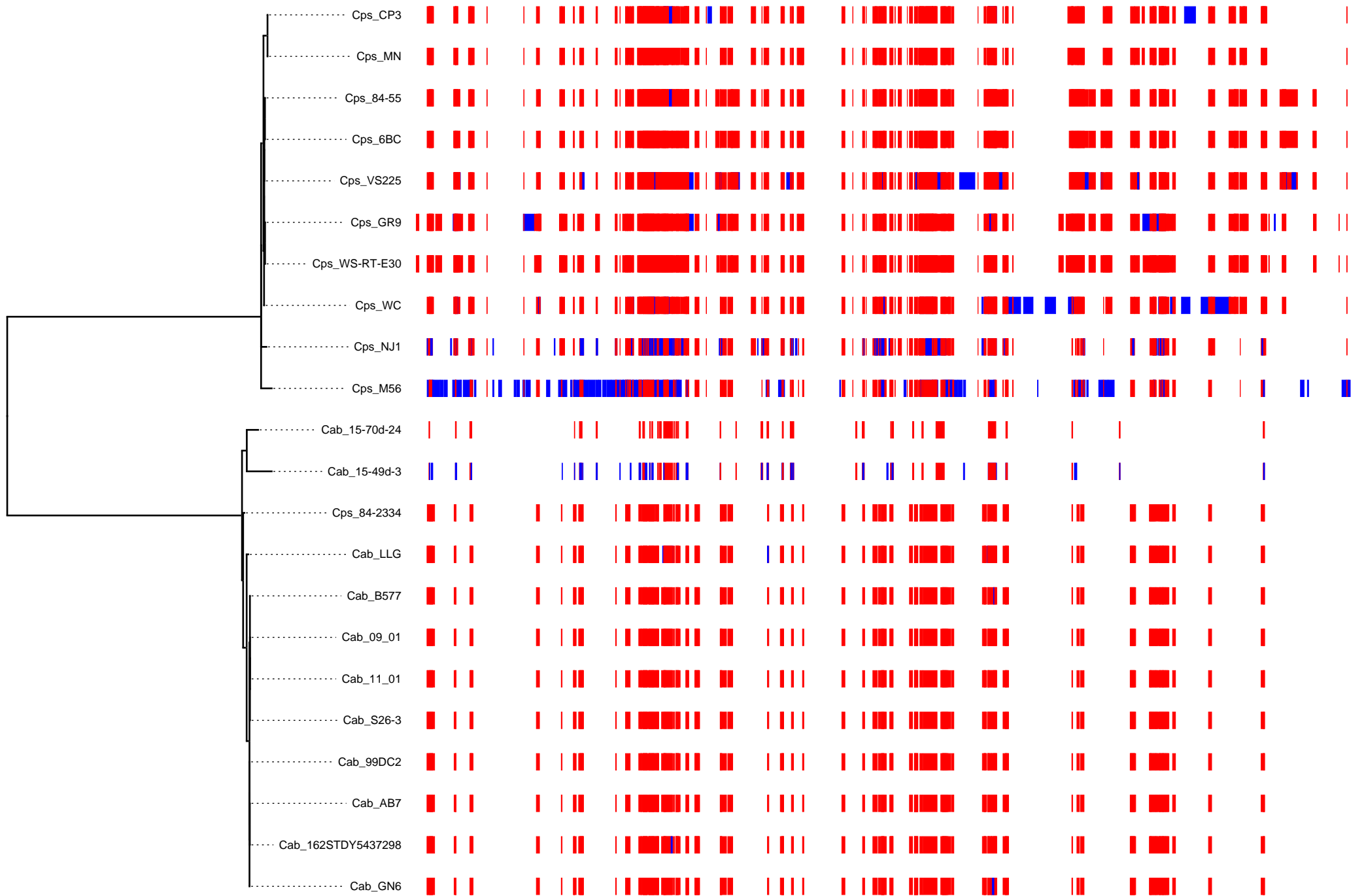

Supplement: Supplementary file 8 — Additional file 8: Fig. S6. Summary of Gubbins recombination analysis including avian C. abortus strains 15-59d/3 and 15-70d/24. The red blocks represent recombination events occurring on an internal branch of the phylogenetic tree, which are shared by several strains by common descent. The blue blocks indicate recombination events occurring on terminal branches of the phylogenetic tree, which are unique to a specific strain. The parameters used for the run are those described in Fig. 4. [file 12864_2021_7477_MOESM8_ESM.pdf]

A

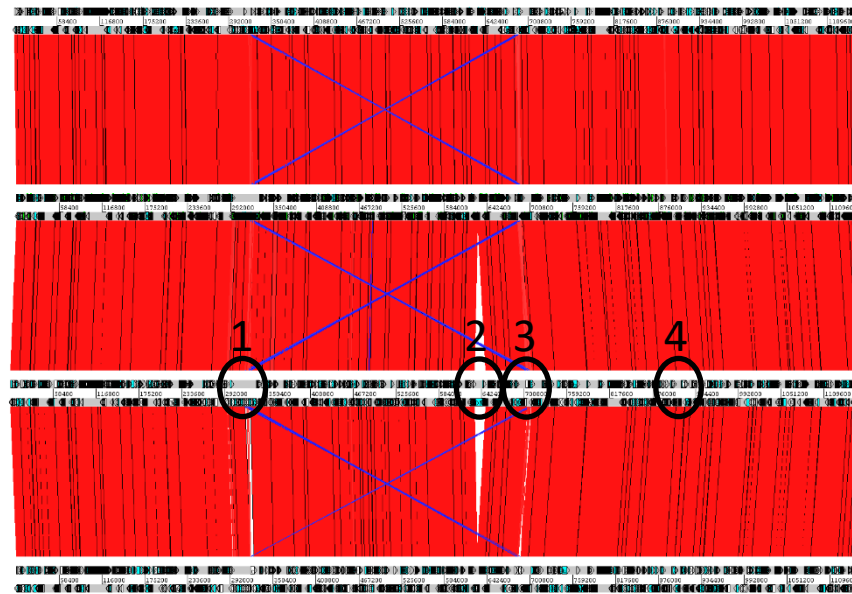

Cab AB7

Cab S26/3

Cps 84/2334

Cab LLG

B

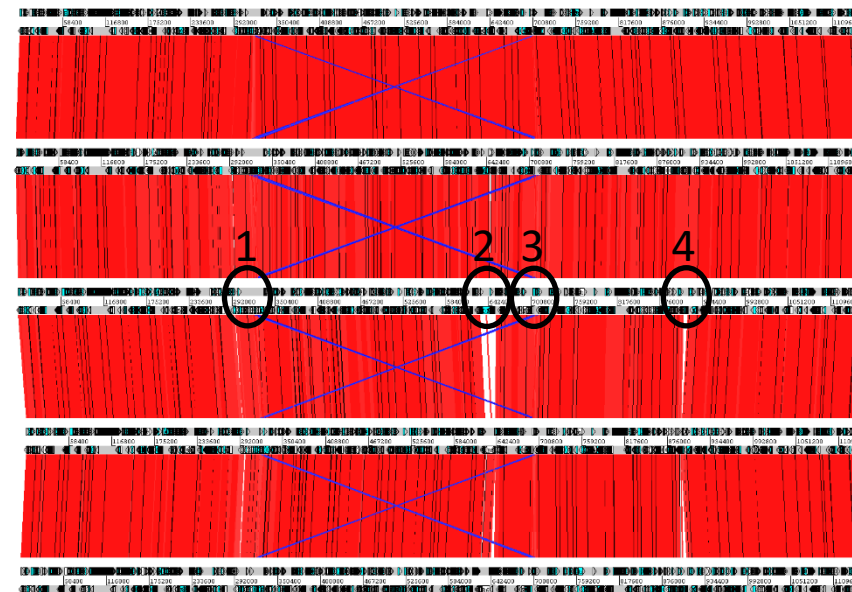

Cps VS225

Cps 84/55

Cps 84/2334

Cps GR9

Cps M56

Supplement: Supplementary file 9 — Additional file 9: Fig. S7. Comparative genome analysis of C. psittaci 84/2334. Whole genome comparisons of C. psittaci strain 84/2334 and (A) C. abortus strains S26/3, AB7 and LLG and (B) representative C. psittaci strains VS225, 84/55, GR9 and M56 depicting amino acid matches computed using Megablast blastn. Homology matches are indicated by the red vertical bars, while inverted matches (indicating areas of recombination) are coloured blue. Horizontal grey bars represent the forward and reverse strands of DNA with CDSs marked as arrows. The main regions of difference occur in two of the Pmp loci (circled areas 1 and 3), the PZ region (circled area 2) and TMH loci (circled area 4). Please note that the gaps shown for C. abortus strain LLG compared to 84/2334 in Pmp loci (circled areas 1 and 3) are due to the sequences not being complete in these regions rather than homology differences. The figure was generated using the Artemis Comparison Tool (ACT). [file 12864_2021_7477_MOESM9_ESM.pdf]

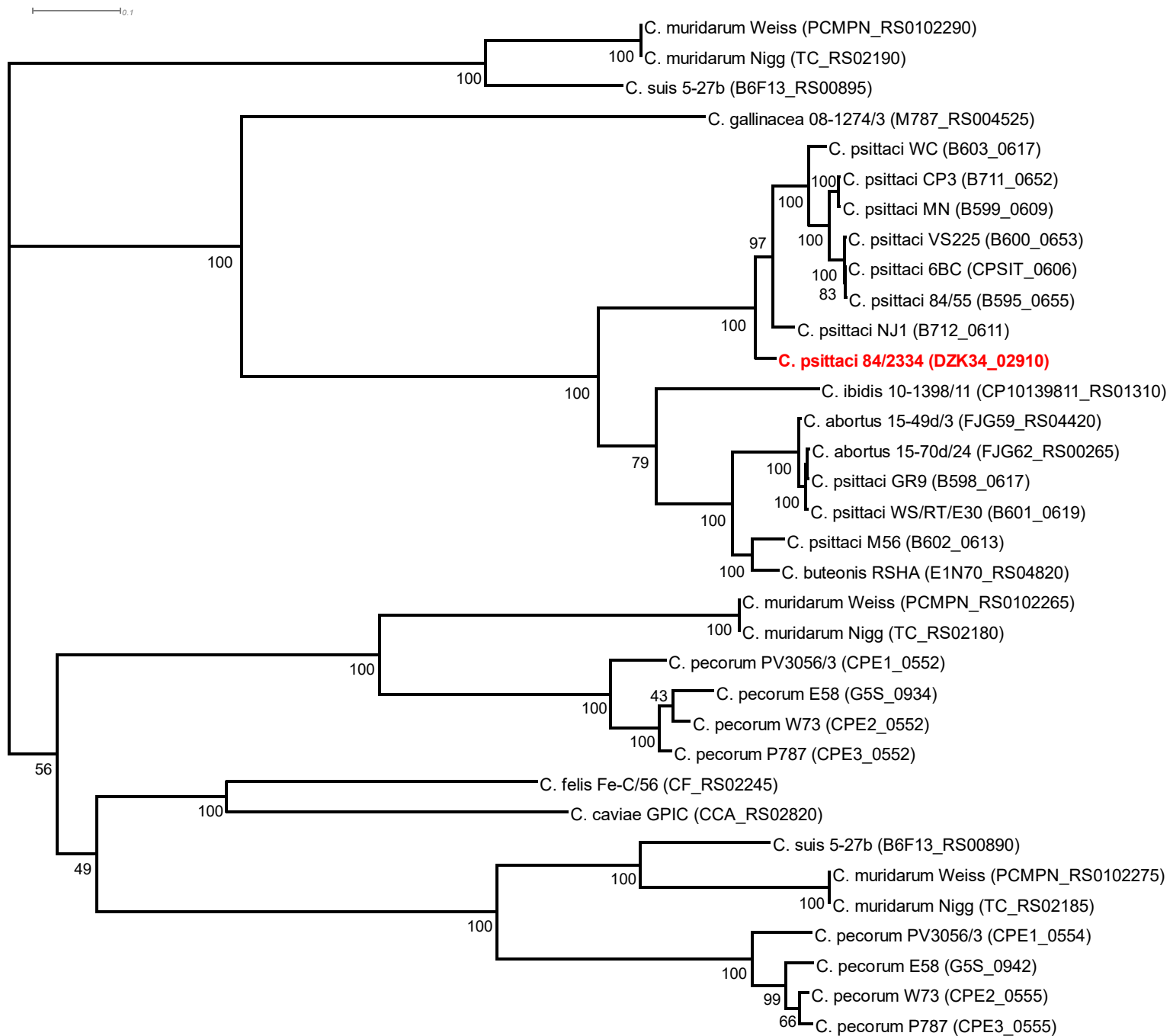

Supplement: Supplementary file 10 — Additional file 10: Fig. S8. Phylogenetic analysis of chlamydial cytotoxin predicted protein sequences. The consensus tree was estimated in IQ-Tree by Maximum Likelihood using a JTTDCMut + F + I + G4 substitution and rate heterogeneity model, according to BIC model selection, and 100 non-parametric bootstrap replicates. The tree is midpoint rooted and bootstrap support is indicated by the number at the node. The scale bar indicates the expected substitutions per site. The tree was prepared in Dendroscope. Strain 84/2334 is in bold and red font. [file 12864_2021_7477_MOESM10_ESM.pdf]

A

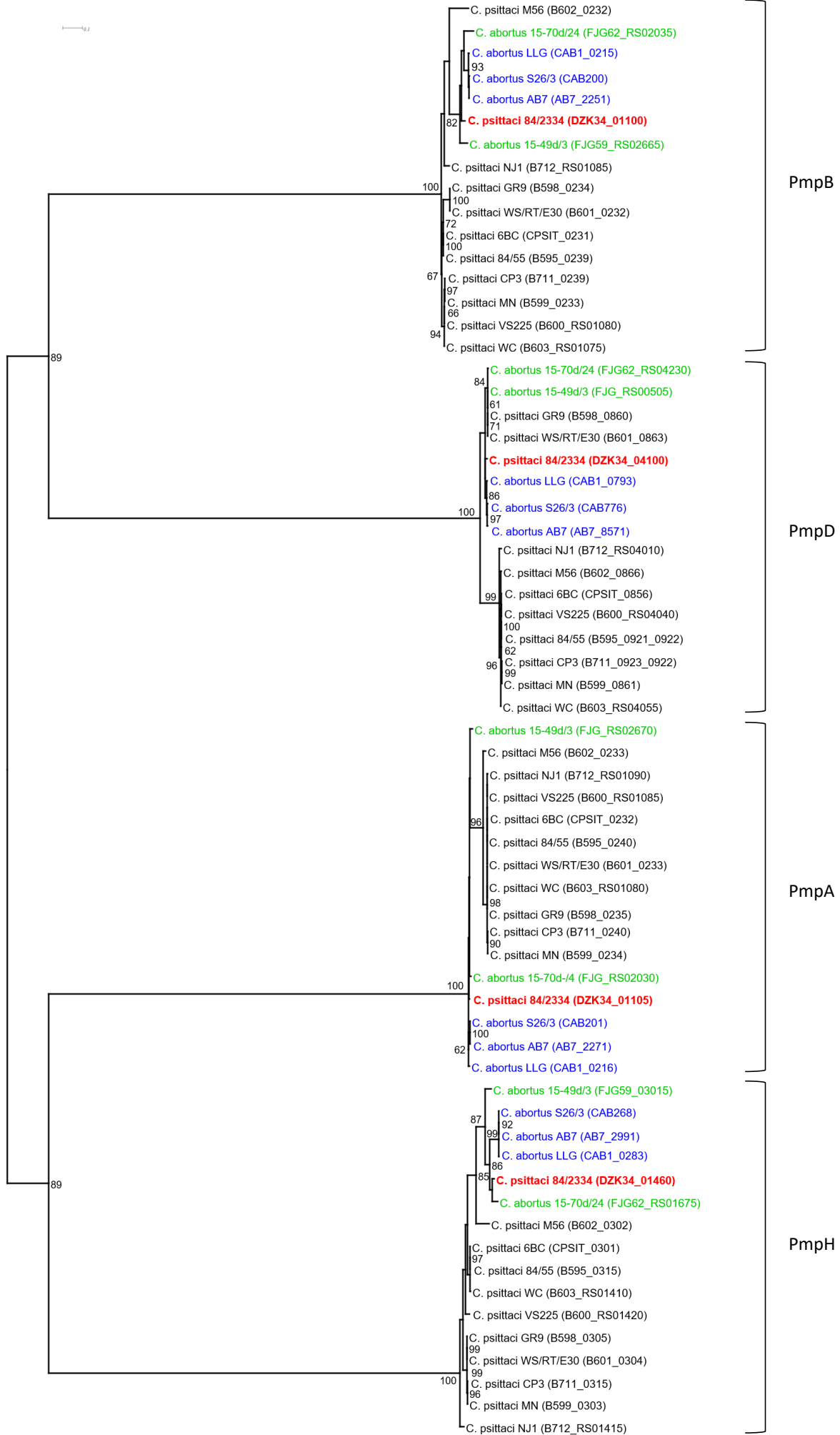

B

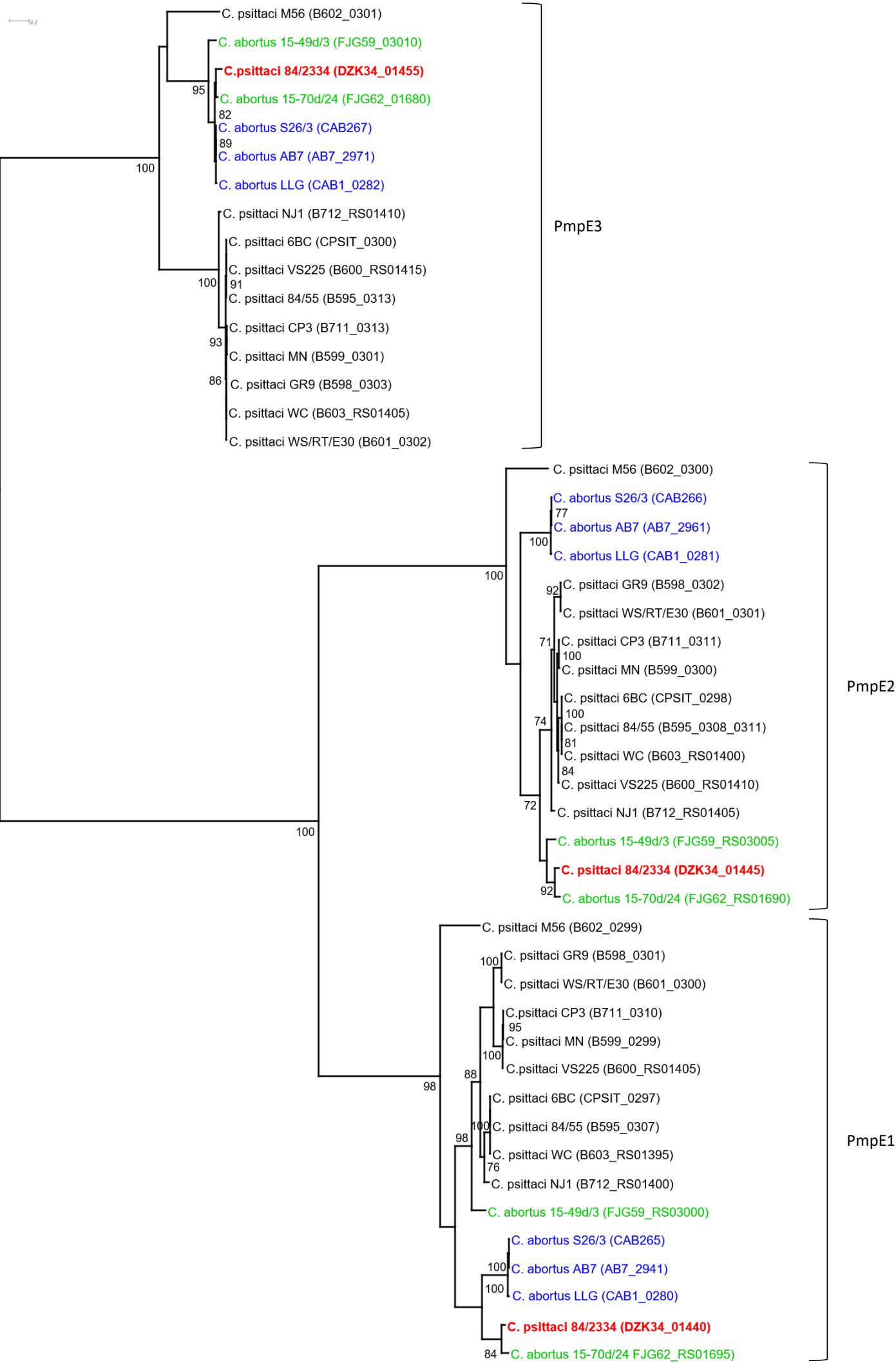

Supplement: Supplementary file 11 — Additional file 11: Fig. S9. Polymorphic membrane protein family phylogenies. Consensus trees for singleton PmpA, PmpB, PmpD and PmpH family members (A), for the PmpE family members (B) and PmpG family members (C) for strain 84/2334 and the C. abortus and C. psittaci strains/genotypes shown in Table 2 were estimated in IQ-Tree by Maximum Likelihood using substitution and rate heterogeneity models JTTDCMut + F + I + G4, JTT + F + R3 and JTT + F + R5, respectively, according to BIC model selection, and 100 non-parametric bootstrap replicates. The trees were midpoint rooted and bootstrap support is indicated by the number at the node (only values greater than 70 are shown). The scale bar indicates the expected substitutions per site. The trees were prepared in Dendroscope. Strain 84/2334 is in bold and red font. Classical and avian C. abortus strains are in blue and green fonts, respectively. [file 12864_2021_7477_MOESM11_ESM.pdf]
