# Supplementary material for: Whole genome de novo sequencing and comparative genomic analyses suggests that Chlamydia psittaci strain 84/2334 should be reclassified as Chlamydia abortus species
Source: BMC Genomics. 2021 Mar 6;22:159. doi: 10.1186/s12864-021-07477-6 (PMC7937271; doi:10.1186/s12864-021-07477-6)
Supplement: Supplementary file 5 — Additional file 5: Fig. S3. Whole genome phylogenetic analysis informed by recombination with added outgroup. Phylogenetic tree of a whole genome sequence MAFFT alignment of the C. abortus (Cab) and C. psittaci (Cps) strains shown in Table 2 as derived by Gubbins after removing genomic regions affected by recombination. Genotypes are given in square brackets. The sequence of C. pecorum W73 was additionally provided as outgroup. FastTree was used as tree builder, and a maximum of 100 iterations was specified in order to guarantee convergence. Tree was midpoint rooted and prepared using Dendroscope. Strain 84/2334 is in bold and red font. Classical and avian C. abortus strains are in blue and green fonts, respectively. [file 12864_2021_7477_MOESM5_ESM.pdf]

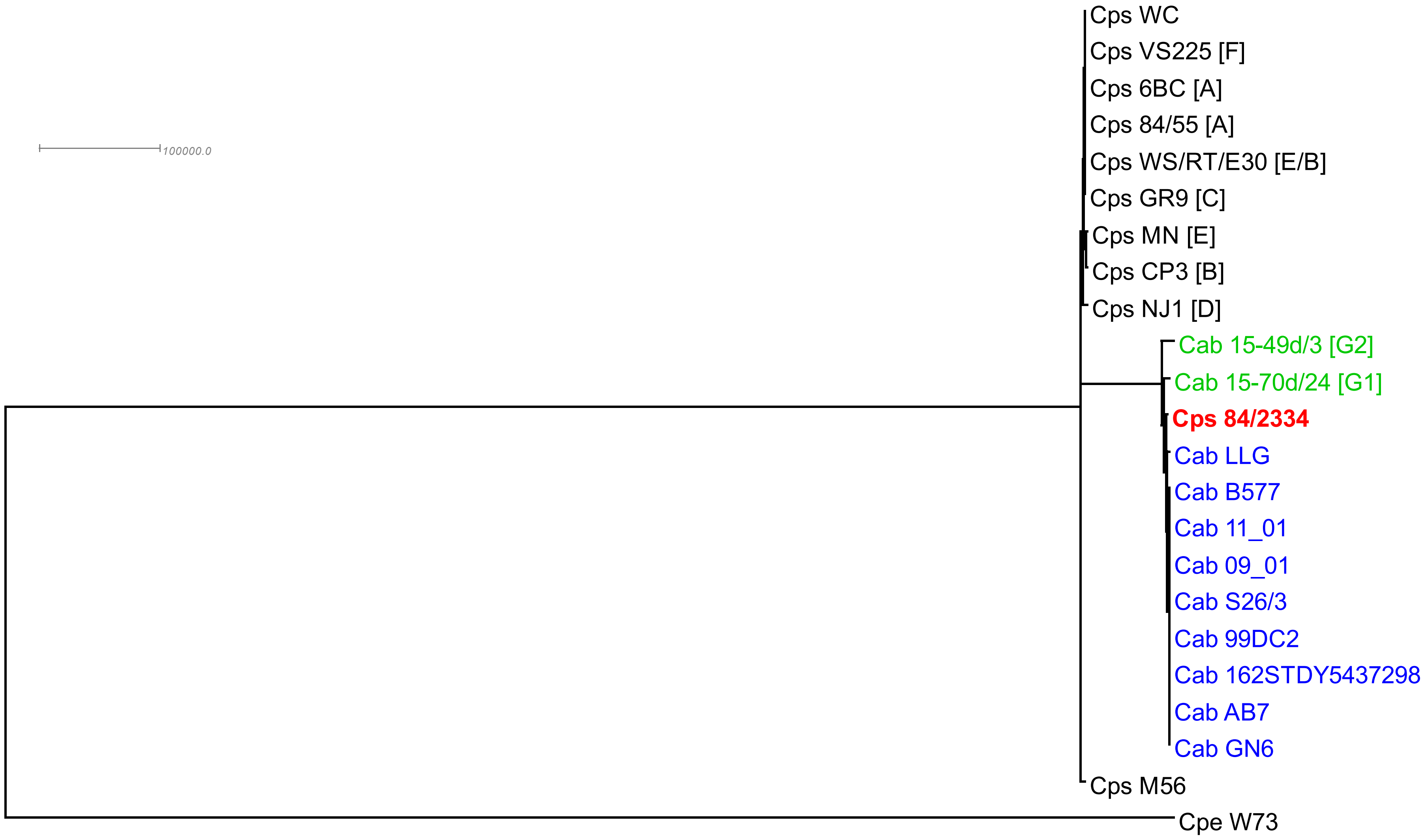
100000.0
Cps WC
Cps VS225 [F]
Cps 6BC [A]
Cps 84/55 [A]
Cps WS/RT/E30 [E/B]
Cps GR9 [C]
Cps MN [E]
Cps CP3 [B]
Cps NJ1 [D]
Cab 15-49d/3 [G2]
Cab 15-70d/24 [G1]
Cps 84/2334
Cab LLG
Cab B577
Cab 11_01
Cab 09_01
Cab S26/3
Cab 99DC2
Cab 162STDY5437298
Cab AB7
Cab GN6
Cps M56
Cpe W73
